# Structural basis of centromeric cohesion protection

**DOI:** 10.1038/s41594-023-00968-y

**Published:** 2023-04-20

**Authors:** Alberto García-Nieto, Amrita Patel, Yan Li, Roel Oldenkamp, Leonardo Feletto, Joshua J. Graham, Laureen Willems, Kyle W. Muir, Daniel Panne, Benjamin D. Rowland

**Affiliations:** 1 Division of Cell Biology, The Netherlands Cancer Institute, Amsterdam, the Netherlands; 2 Leicester Institute of Structural and Chemical Biology, Department of Molecular and Cell Biology, University of Leicester, Leicester, UK; 3 Department of Fundamental Microbiology, Faculty of Biology and Medicine, University of Lausanne, Lausanne, Switzerland; 4 MRC Laboratory of Molecular Biology, Cambridge, UK

**Keywords:** Centromeres, X-ray crystallography, Cohesion, Centromeres, Cohesion

## Abstract

In the early stages of mitosis, cohesin is released from chromosome arms but not from centromeres. The protection of centromeric cohesin by SGO1 maintains the sister chromatid cohesion that resists the pulling forces of microtubules until all chromosomes are attached in a bipolar manner to the mitotic spindle. Here we present the X-ray crystal structure of a segment of human SGO1 bound to a conserved surface of the cohesin complex. SGO1 binds to a composite interface formed by the SA2 and SCC1^RAD21^ subunits of cohesin. SGO1 shares this binding interface with CTCF, indicating that these distinct chromosomal regulators control cohesin through a universal principle. This interaction is essential for the localization of SGO1 to centromeres and protects centromeric cohesin against WAPL-mediated cohesin release. SGO1–cohesin binding is maintained until the formation of microtubule–kinetochore attachments and is required for faithful chromosome segregation and the maintenance of a stable karyotype.

## Abstract (web summary)

Here, the authors determine the structural basis of how SGO1 protects centromeric cohesin from WAPL-mediated release until the kinetochores and the microtubules have formed functional attachments. In addition, they show that SGO1 and CTCF bind cohesin in a similar manner, suggesting a potential universal mechanism of cohesin control.

## Main

During mitosis, the duplicated genome needs to be accurately distributed over the two daughter cells. The cohesin protein complex holds together the sister DNAs from replication until mitosis^1–3^. Cohesin entraps DNA inside its ring-shaped structure^4^, which at its core consists of SMC1, SMC3 and SCC1 (also known as RAD21 or Mcd1). SCC1 is bound by either of two paralogous HEAT repeat-containing proteins, SA1 or SA2 (also known as STAG1 and STAG2)^5^.

Cohesin complexes have a dynamic mode of DNA binding that involves DNA entrapment and release. From DNA replication until mitosis, the cohesin complexes that hold together the sister DNAs are locked on DNA to render cohesin resistant to cohesin’s release factor WAPL^6^. During mitosis, cohesin is removed from chromosomes in two waves. First, cohesin is removed from chromosome arms in a WAPL-dependent manner through a process known as the prophase pathway^7–10^. Cohesion at centromeres is protected by Shugoshin (SGO1)^11–14^, giving rise to the typical X-shaped structure of human chromosomes. SGO1 protects centromeric cohesin by recruiting PP2A to counteract cohesin phosphorylation by mitotic kinases, and SGO1 also directly competes with WAPL for cohesin binding^15–18^. Centromeric cohesion is maintained until proper attachment of microtubules to the kinetochores, upon which the remaining cohesin is cleaved by separase to trigger anaphase onset^19^. By protecting centromeric cohesion, SGO1 thus ensures faithful chromosome segregation.

Cohesin has a dual role, as it not only holds together sister DNAs but also builds the DNA loops that shape the interphase genome. To control this latter function, cohesin is bound by the architectural factor CTCF^20^. We showed recently that CTCF binds to cohesin through a conserved YxF motif in the amino terminus of CTCF. This CTCF segment interacts directly with a composite binding interface formed by the SA2 and SCC1 subunits of cohesin^21^. The SA2 interface is conserved from fungi to mammals and is known as the conserved essential surface (CES)^22^. For simplicity, we refer to the composite SA2–SCC1 binding pocket as the CES. The direct interaction of the YxF motif of CTCF with the CES is required for formation of CTCF-anchored loops at TAD boundaries^21^. It has also been suggested that the CES region interacts directly with both SGO1 and WAPL^18^. The interaction of SGO1 with cohesin is promoted by phosphorylation of SGO1 T346. However, this phosphorylation does not control the direct interaction between the CES and SGO1 (refs. ^17,18^). SGO1 contains a YxF motif close to this phosphorylation site that could be key to this interaction. In agreement, previous biochemical experiments have shown that an SGO1 fragment containing the YxF motif can directly compete with CTCF for binding to the CES^21^. If so, cohesin complexes may be controlled through a shared mechanism, irrespective of whether these complexes build DNA loops or rather hold together the sister DNAs.

**Fig. 1:**
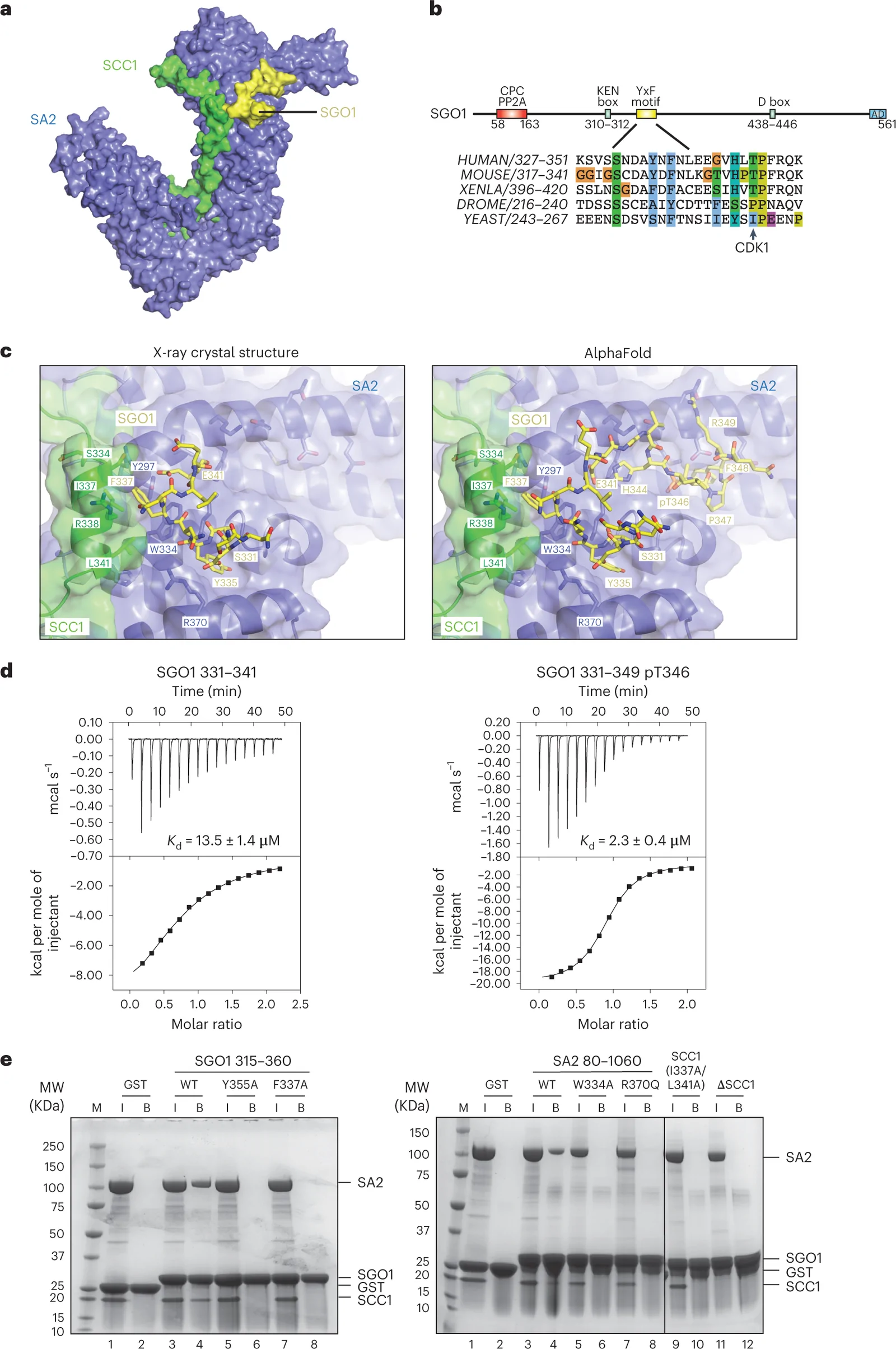
Structure of the SA2–SCC1–SGO1 complex. **a**, Structure of the SA2–SCC1–SGO1 complex. SA2 (blue), SCC1 (green) and SGO1 (yellow). **b**, Domain architecture and conservation of the YxF motif in SGO1. **c**, Crystal structure (left) and AlphaFold model (right) of the SA2–SCC1–SGO1 complex. Details of the CES binding pocket showing the interactions of SGO1 F337 and Y335. AlphaFold model: SGO1 amino acids spanning 341–349 including pT346 are predicted to form additional interactions with SA2. **d**, ITC experiments with SGO1 amino acids 331–341 SNDAYNFNLEE (left) and 331–349 SNDAYNFNLEEGVHLpTPFR containing phosphorylated pT346 (right). **e**, GST pulldown analysis of SGO1 and SA2 or SCC1 variants. M, molecular weight marker; I, input; B, bound fractions, analyzed by SDS polyacrylamide gel electrophoresis. Controls are shown in lanes 1 and 2. The experiment was repeated three times with consistency. WT, wild type.

We report here the X-ray crystal structure and AlphaFold model of the cohesin subcomplex SA2–SCC1 bound to a fragment of SGO1 (Fig. 1a). We demonstrate that SGO1 engages the CES of SA2–SCC1 through its YxF motif. The binding mode is similar to that seen with the YxF motif from CTCF. Mutations that abolish SGO1–CES interaction interfere with the localization of SGO1 to centromeres and lead to severe cohesion defects. We infer that engagement of the CES surface of cohesin by distinct chromosomal regulators is a universal principle that allows control of cohesin function during different chromosomal processes.

## Results

### Structural basis for SGO1–cohesin interaction

**Extended Data Fig. 1:**
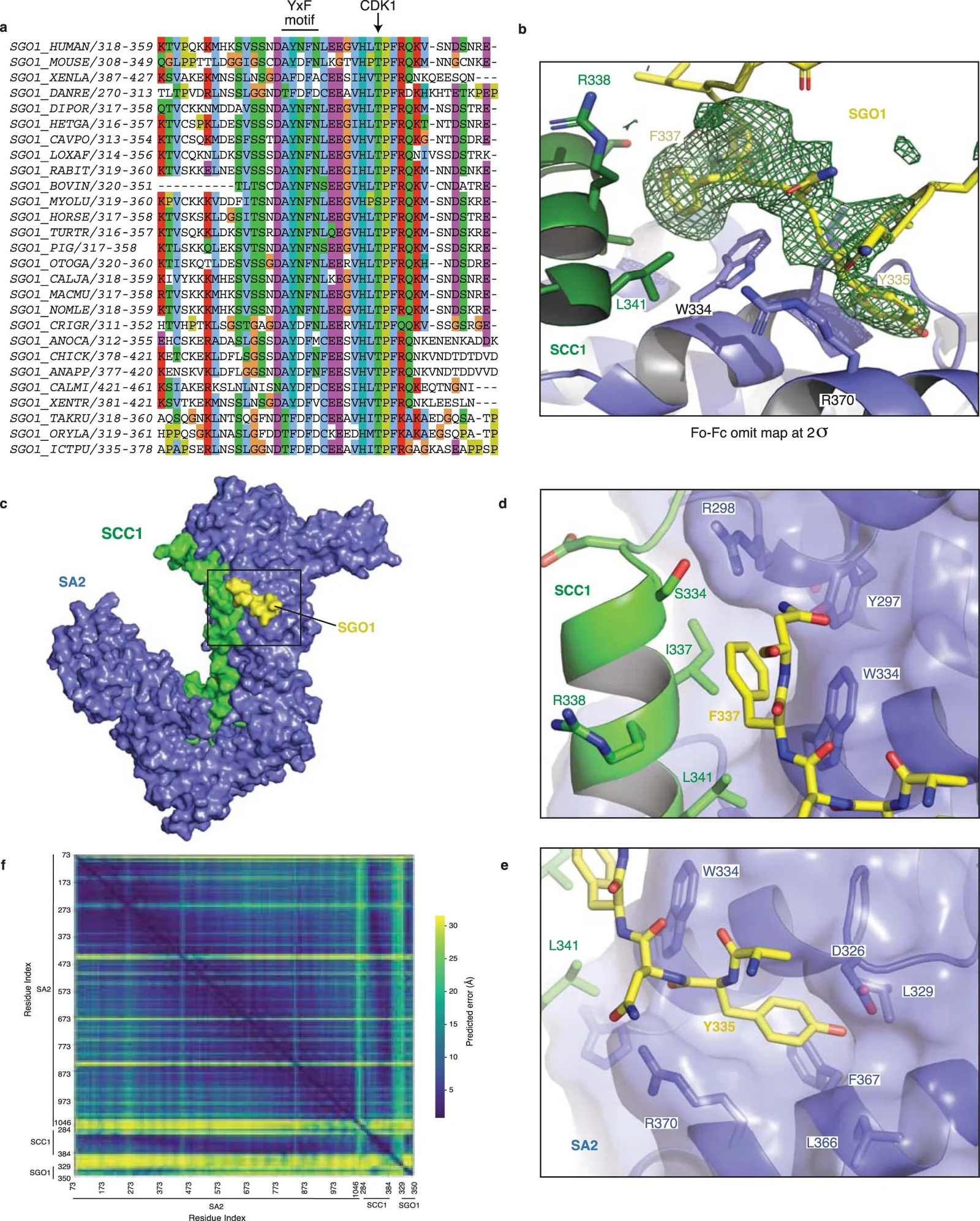
Structural analysis of the SA2-SCC1-SGO1 complex. **a**, Conservation of the YxF motif and CDK1 T346 phosphorylation site in SGO1 in vertebrates. **b**, Fo − Fc omit electron-density Fourier map contoured at 2σ. **c**, Surface-rendered cartoon of the SA2-SCC1-SGO1 complex. **d**, Details of the SA2-SCC1 binding pocket for SGO1 F337 or **e**, Y335. **f**, AlphaFold: Predicted Aligned Error (PAE) plot for each amino acid residue in the SA2-SCC1-SGO1 complex.

Previous data indicate that SGO1 interacts directly with the SA2–SCC1 subunit of cohesin^18^. The interacting region contains a YxF motif that is conserved in vertebrate SGO1 proteins (Fig. 1b and Extended Data Fig. 1a). Phosphorylation of T346, probably by CDK1 (ref. ^17^), enhances the interaction with SA2–SCC1 but is not essential for binding^18^. We were able to obtain crystals with a SGO1 peptide spanning amino acids 331–341 containing the YxF motif but not with an extended peptide spanning amino acids 331–349 containing pT346. We determined the X-ray structure by molecular replacement to a minimum Bragg spacing of 3.2 Å (Table 1). An Fo–Fc omit electron-density Fourier map exhibited clear features of the SGO1 peptide (Extended Data Fig. 1b). The SGO1 peptide is bound to the CES binding pocket (Fig. 1c and Extended Data Fig. 1c–e). Amino acid residues F337 and Y335 of SGO1 bind into hydrophobic pockets using a similar binding mode to that seen previously for CTCF^21^. Briefly, the binding pocket for F337 of SGO1 contains amino acids S334, I337 and L341 from SCC1 and Y297 and W334 from SA2 (Fig. 1c and Extended Data Fig. 1d). Y335 of SGO1 binds in a deep hydrophobic pocket containing L329, L366 and F367 (Fig. 1c and Extended Data Fig. 1e). A model calculated using AlphaFold structure prediction^23^ showed an almost identical binding mode and suggested additional interactions between SGO1 amino acids 341–349 and SA2 (Fig. 1c).

**Table 1:** X-ray data collection and refinement statistics

	SA2–SCC1–SGO1 (PDB 7ZJS)
**Data collection**	
Space group	P2_1_
Cell dimensions	
*a*, *b*, *c* (Å)	78.80, 181.09, 111.37
α, β, γ (°)	90, 94.25, 90
Resolution (Å)	47.8–3.24 (3.35–3.24)^*^
*R*_sym_ or *R*_merge_	8.76 (116)^*^
*I*/σ *I*	8.1 (0.74)^*^
*CC 1*/*2*	0.99 (0.45)^*^
Completeness (%)	99.8 (90.71)^*^
Multiplicity	2.7 (2.7)^*^
**Refinement**	
Resolution (Å)	47.8–3.24
*R*_work_/*R*_free_	0.25/0.28
Unique reflections	48451 (4517)
No. atoms	16119
SA2	14692
SCC1	1192
SGO1	135
*B* factors (mean; Å^2^)	
SA2	114.6
SCC1	99.6
SGO1	118.1
R.m.s deviations	
Bond lengths (Å)	0.008
Bond angles (°)	1.12

^*^Values in parentheses are for the highest-resolution shell.

Accordingly, isothermal titration calorimetry (ITC) experiments showed that the T346-phosphorylated SGO1 fragment spanning amino acids 331–349 bound SA2–SCC1 with a lower equilibrium dissociation constant (2.3 ± 0.4 μM) compared with a nonphosphorylated SGO1 peptide spanning amino acids 331–341 (13.5 μM ± 1.4) (Fig. 1d). Using glutathione S-transferase (GST) pulldown experiments, we found that SGO1 retained SA2–SCC1 on GST beads (Fig. 1e). Mutation Y335A or F337A of SGO1 abolished the interaction. Mutation of critical CES amino acid residues including SA2 W334A, R370Q, SCC1 I337A L341A or the absence of SCC1 also impaired SGO1 binding. Together, our data confirm the previous biochemical mapping of SGO1 interaction^18^. We conclude that the YxF motif of SGO1 is essential for binding to the composite CES of SA2–SCC1. Phosphorylation of SGO1 at T346 enhances the interaction.

### The SGO1–CES interaction protects centromeric cohesion

**Fig. 2:**
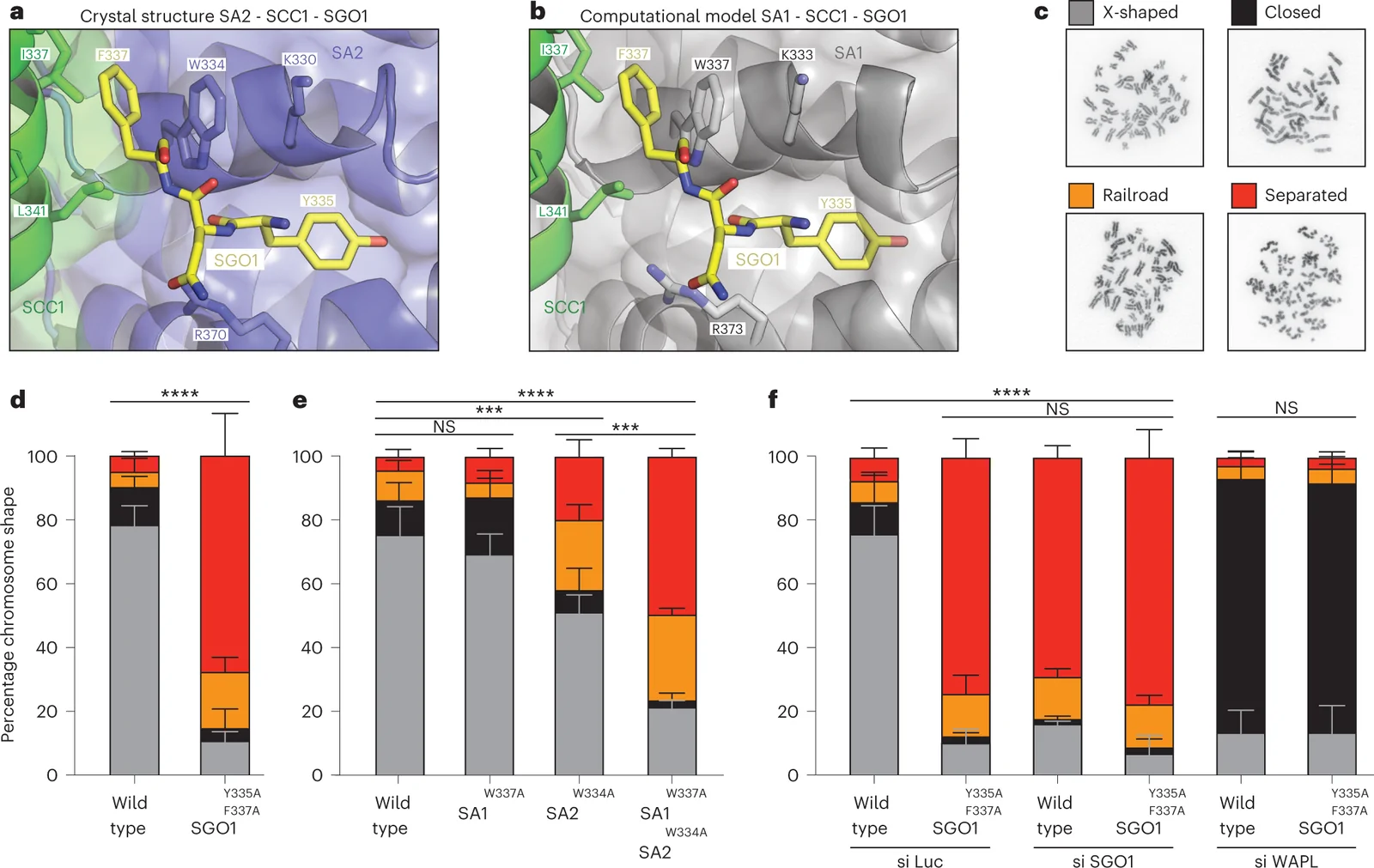
The SGO1–CES interaction protects centromeric sister chromatid cohesion. **a**, Zoomed-in view of the CES binding pocket of SA2 (blue) and SCC1 (green) bound to SGO1 (yellow). **b**, Computational model containing SA1 (gray). **c**, Representative images of different chromosome phenotypes during prometaphase. **d**, Quantification of chromosome phenotypes in prometaphase wild-type cells and SGO1^Y335A F337A^ cells (unpaired *t* test; ****P < 0.0001). **e**, Quantification of the chromosome phenotypes in prometaphase wild-type, SA1^W337A^, SA2^W334A^ and SA1^W337A^ SA2^W334A^ cells (unpaired *t* test; ***P ≤ 0.0004, ****P < 0.0001, NS, not significant). **f**, Quantification of chromosome phenotypes in prometaphase wild-type and SGO1^Y335A F337A^ cells after treatment with either siLuciferase (siLuc), siSGO1 or siWAPL. All panels depict the mean ± s.d. of three independent experiments with more than 70 cells analyzed per experiment (unpaired *t* test; ****P < 0.0001).

**Extended Data Fig. 2:**
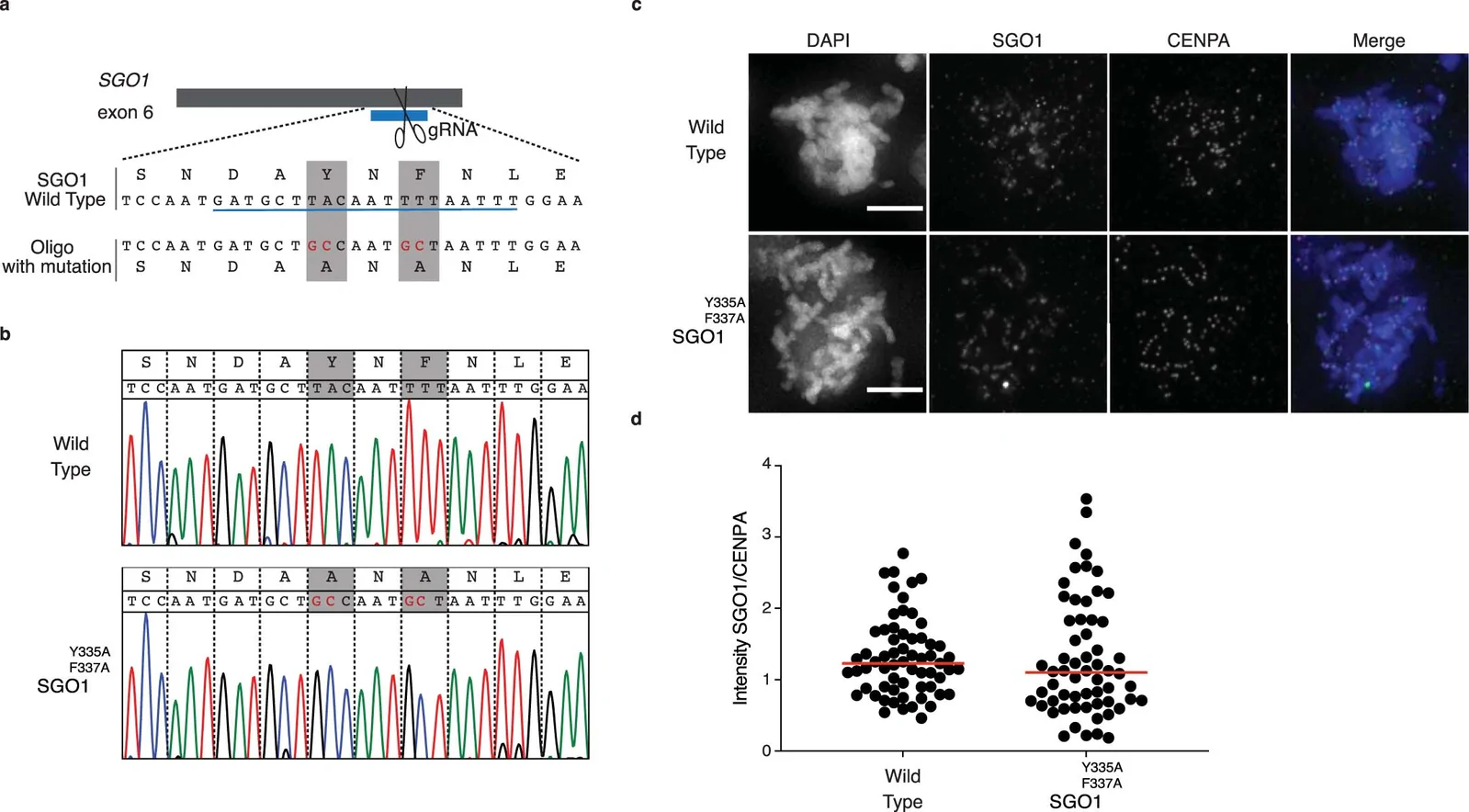
Generation of SGO1 Y335A/F337A cells. **a**, Schematic of CRISPR-Cas9 mediated generation of SGO1 Y335A/F337A cells. Cleavage of exon 6 of the *SGO1* gene in the presence of a repair oligonucleotide that includes mutant codons encoding Y335A and F337A. **b**, The SGO1 Y335A/F337A mutation was confirmed by Sanger sequencing. **c**, Expression of endogenous SGO1 (Green) and CENPA (Magenta) in prometaphase wild type and SGO1 Y335A/F337A cells. Representative image of three independent experiments. Scale-bar: 5 μm. **d**, Quantification of SGO1 levels, normalized to CENPA levels, in prometaphase wild type and SGO1 Y335A/F337A cells. Median in red of a representative graph of three independent experiments, with over 60 prometaphase cells analysed per experiment. Source data

**Extended Data Fig. 3:**
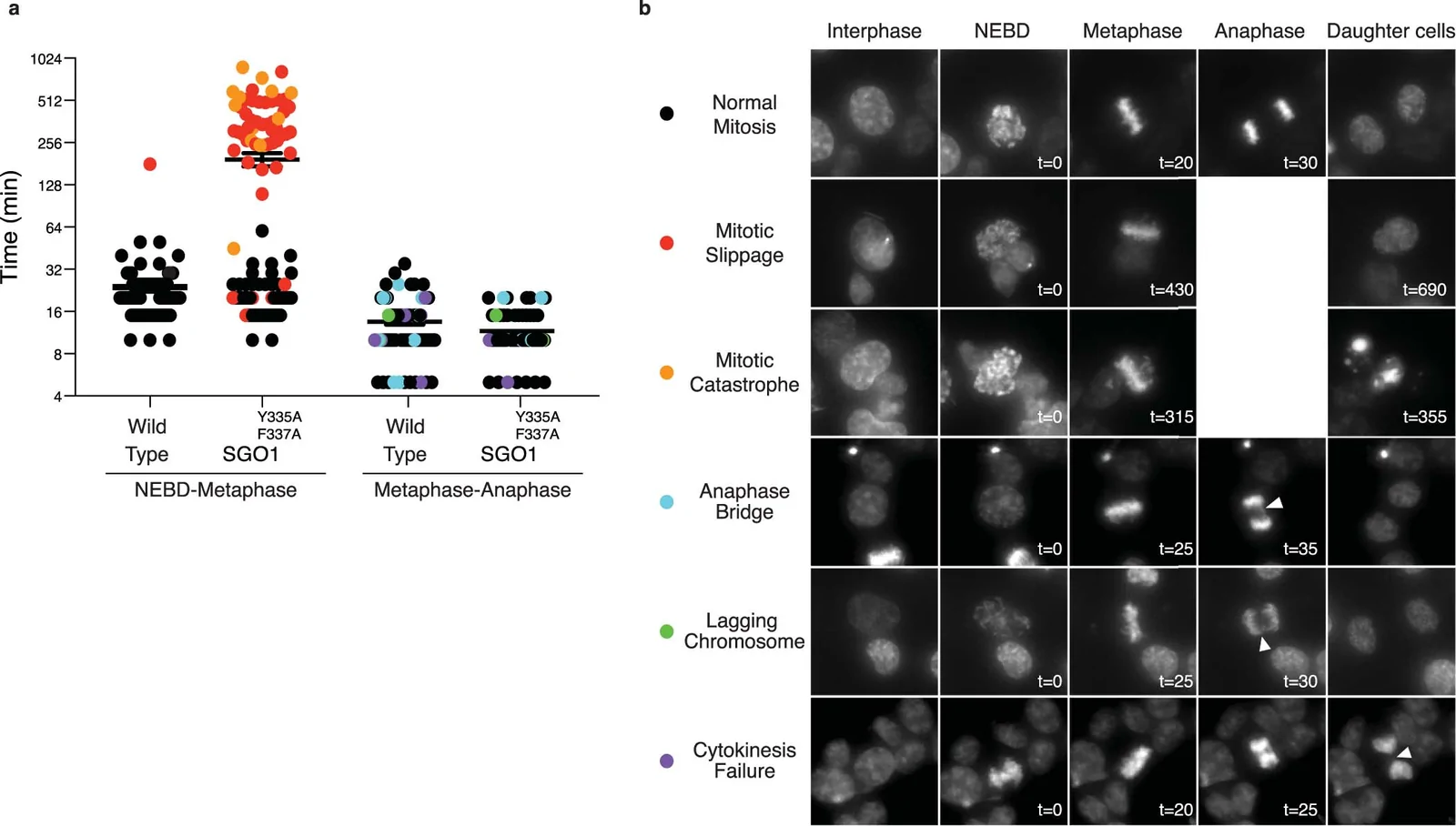
SGO1 Y335A/F337A cells display mitotic delay. **a**, Time in mitosis from Nuclear Envelop Breakdown (NEBD) to metaphase, and from metaphase to anaphase in wild type and SGO1 sY335A/F337A cells. Mean +/− SD of a representative graph of two independent experiments with over 100 mitotic cells analysed. **b**, Types of mitotic progression defects for the cells as timed in **(a)** including representative images of the progression through mitosis. Time is depicted in minutes. Arrowheads mark the segregation defect observed upon anaphase onset. Source data

To test whether the SGO1–cohesin interaction that we identified in our crystal structure controls sister chromatid cohesion, we mutated the endogenous SGO1 allele in HAP1 cells using CRISPR–Cas9 technology. We thereby obtained HAP1 cells with SGO1^Y335A F337A^ as their sole copy of SGO1 (Extended Data Fig. 2a–d). We then analyzed sister chromatid cohesion in these cells by performing chromosome spreads. Wild-type cells, as expected, displayed robust sister chromatid cohesion. SGO1^Y335A F337A^ cells, however, displayed severe cohesion defects (Fig. 2c,d). Correspondingly, a large proportion of these cells failed to form a proper metaphase plate, leading either to mitotic slippage or mitotic catastrophe (Extended Data Fig. 3a,b).

**Extended Data Fig. 4:**
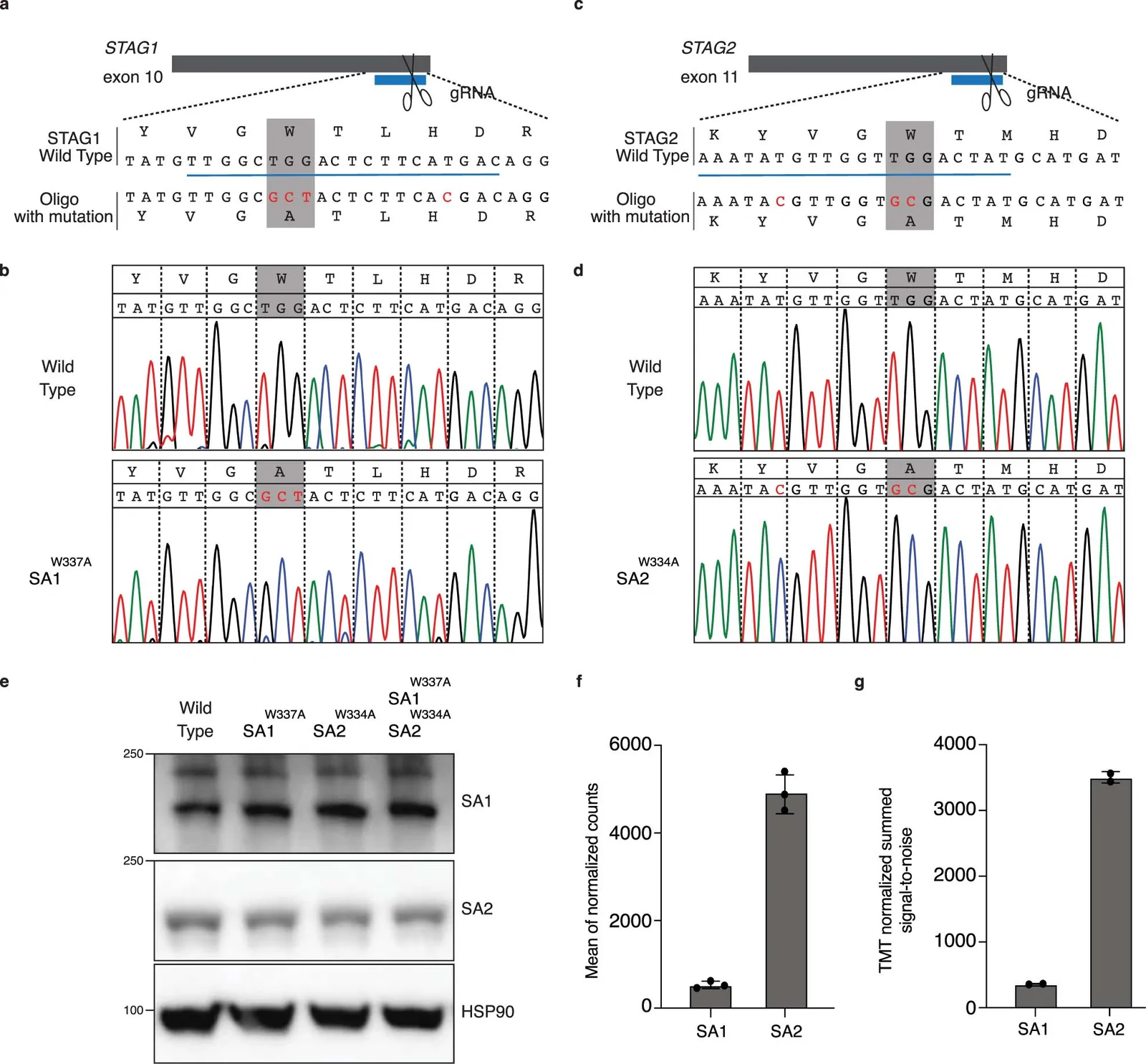
Generation of CES mutant cells. **a**, Schematic of CRISPR-Cas9 mediated generation of SA1 W337A cells. Cleavage of exon 10 of the *STAG1* gene in the presence of a repair oligonucleotide that includes mutant codon encoding SA1 W337A. **b**, The SA1 W337A mutation was confirmed by Sanger sequencing. **c**, Schematic of CRISPR-Cas9 mediated generation of SA2 W334A cells, using the approach of panel **(a)**, but now targeting exon 11 of *STAG2* gene and using the repair oligo encoding SA2 W334A. **d**, The SA2 W334A mutation was confirmed by Sanger sequencing, in addition to a silent mutation. **e**, Representative western blot of HAP1 wild type, SA1 W337A, SA2 W334A, and SA1 W337A/SA2 W334A cells. HSP90 serves as a loading control. Experiment performed two times **f**, RNA levels of SA1 and SA2 in wild type HAP1 cells (data from^21^). Mean +/− SD of three independent experiments **g**, Protein expression of SA1 and SA2 in wild type HAP1 cells (data from^24^). Mean +/− SD of two independent experiments. Source data

**Extended Data Fig. 5:**
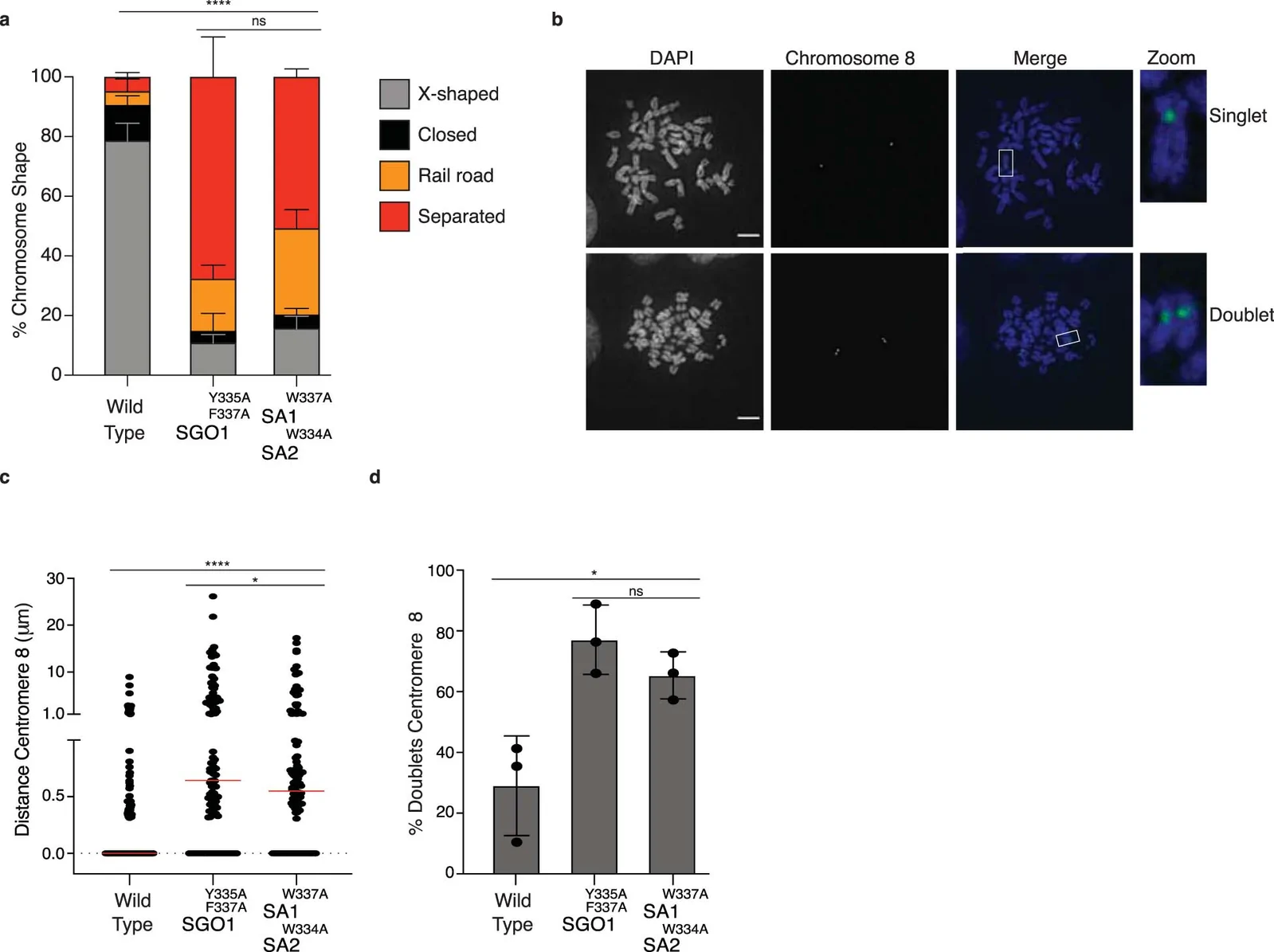
Similar cohesion defects in SGO1 Y335A/F337A and SA1 W337A/SA2 W334A cells. **a**, Comparison of the cohesion defects of SGO1 Y335A/F337A to SA1 W337A/SA2 W334A cells. Wild type and the SGO1 mutant depict the same data as Fig. 2d. Mean +/− SD of three independent experiments, with over 70 cells analysed per experiment (Unpaired T-test; ****P <0.0001; ns, not significant). **b**, Representative images of prometaphase cells, using a FISH probe for the centromere of chromosome 8 (green). Centromeric cohesion was categorized into singlets when the measured distance between fluorescent dots was ≤300 nm, and doublets when the distance was >300 nm. Scale-bar: 5 μm **c**, Representative graph of the distance between fluorescent dots of centromeres of chromosome 8 in wild type, SGO1 Y335A/F337A, and SA1 W337A/SA2 W334A cells. (Unpaired T-test; ****P <0.0001; *P= 0.045). We analysed a minimum of 120 fluorescent events per condition. Three independent replicate experiments were performed. Median shown in red. **d**, Percentage of doublets for wild type, SGO1 Y335A/F337A, and SA1 W337A/SA2 W334A cells. We analysed a minimum of 120 fluorescent events per condition. Three independent replicate experiments were performed. Mean +/− SD are depicted (Unpaired T-test; * *P*=0.014; ns, not significant). Source data

**Extended Data Fig. 6:**
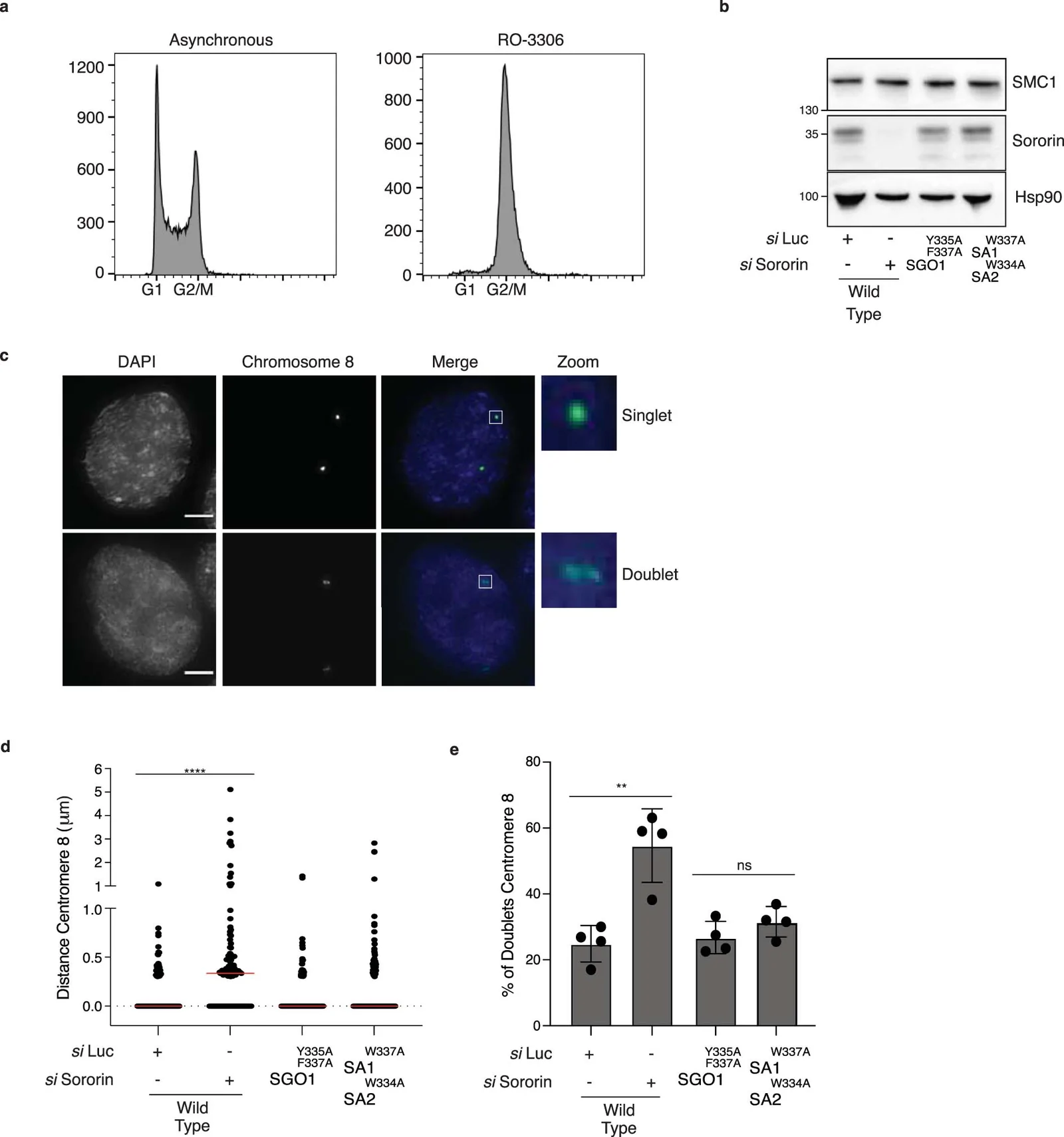
The SGO1/CES interaction does not affect G2 centromeric cohesion. **a**, DNA content of asynchronously dividing cells or of cells treated with the CDK1 inhibitor, RO-3306. **b**, Representative western blots for wild type, SGO1 Y335A/F337A, and SA1 W337A/SA2 W334A cells treated with RO-3306, and of siSororin treated control cells. Hsp90 serves as loading control. Experiment performed three independent times **c**, Representative image of G2 cells using a FISH probe for the centromere of chromosome 8 (green). The centromeres were categorized into singlets when the measured distance between fluorescent dots was ≤ 300 nm, and doublets when the distance was >300 nm. Scale-bar: 5 μm **d**, Representative graph of the distance between fluorescent dots of centromeres of chromosome 8 in wild type, siSororin treated, SGO1 Y335A/F337A, and SA1 W337A/SA2 W334A cells. We analysed a minimum of 120 fluorescent events per condition. Four independent replicate experiments were performed. Median depicted in red (Unpaired T-test, ****P<0.0001). **e**, Percentage of doublets for wild type, siSororin treated, SGO1 Y335A/F337A, and SA1 W337A/SA2 W334A cells We analysed a minimum of 120 fluorescent events per condition. Four independent replicate experiments were performed. Mean +/− SD are depicted. (Unpaired T-test; **P= 0.031; ns, not significant). Source data

Next, we mutated the SGO1 binding interface on cohesin. This CES interface is conserved in both SA1 and SA2 (Fig. 2a,b). We therefore first investigated the relative contributions of each of these SGO1 binding interfaces. We thus mutated SA2 W334A and the equivalent amino acid residue W337A in SA1 (Extended Data Fig. 4a–e). In the SA2–SCC1–SGO1 crystal structure, as well as in the computational model containing SA1, this amino acid was sandwiched between Y335 and F337 of SGO1 (Fig. 2a,b). We found that SA1^W337A^ and SA2^W334A^ cells displayed different phenotypes (Fig. 2e). Whereas sister chromatid cohesion appeared to be unaffected in SA1^W337A^ cells, the SA2^W334A^ cells displayed clear cohesion defects. This indicates that the SGO1–SA2 interaction is more important for cohesion than the SGO1–SA1 interaction. A possible explanation for the observed difference between SA1 and SA2 is the relative abundance of each subunit, as SA2 is approximately ten times more abundant than SA1 in HAP1 cells (Extended Data Fig. 4f,g)^21,24^. Notably, the SA2^W334A^ phenotype was less dramatic than that of SGO1^Y335A F337A^. To test whether wild-type SA1 might compensate for mutation of SA2, we then generated SA1^W337A^ SA2^W334A^ double-mutant cells. These cells indeed displayed cohesion defects that were more severe than those of the SA2^W334A^ single mutant and were similar to those of the SGO1^Y335A F337A^ mutant (Fig. 2e and Extended Data Fig. 5a). With fluorescence in situ hybridization (FISH) experiments, we found no evident role for the SGO1–CES interaction in G2 cohesion (Extended Data Fig. 6 and Supplementary Figure 1). Together, these results indicate that the SGO1–CES interaction plays a crucial part in mitotic sister chromatid cohesion.

### CES binding is a main role of SGO1 and protects against WAPL

To investigate the contribution of the SGO1–CES interaction to SGO1-dependent cohesin protection, we compared the cohesion defects of SGO1^Y335A F337A^ cells with those of cells in which SGO1 was depleted by short interfering RNAs (siRNAs). As expected, this SGO1 depletion yielded a massive cohesion defect, but this defect was no stronger than the defect observed in the SGO1^Y335A F337A^ cells. SGO1 depletion in SGO1^Y335A F337A^ cells also barely if at all worsened the cohesion defect of these cells (Fig. 2f). Together, these results suggest that the SGO1–CES interaction represents an important if not the main role of SGO1 in cohesin protection.

**Extended Data Fig. 7:**
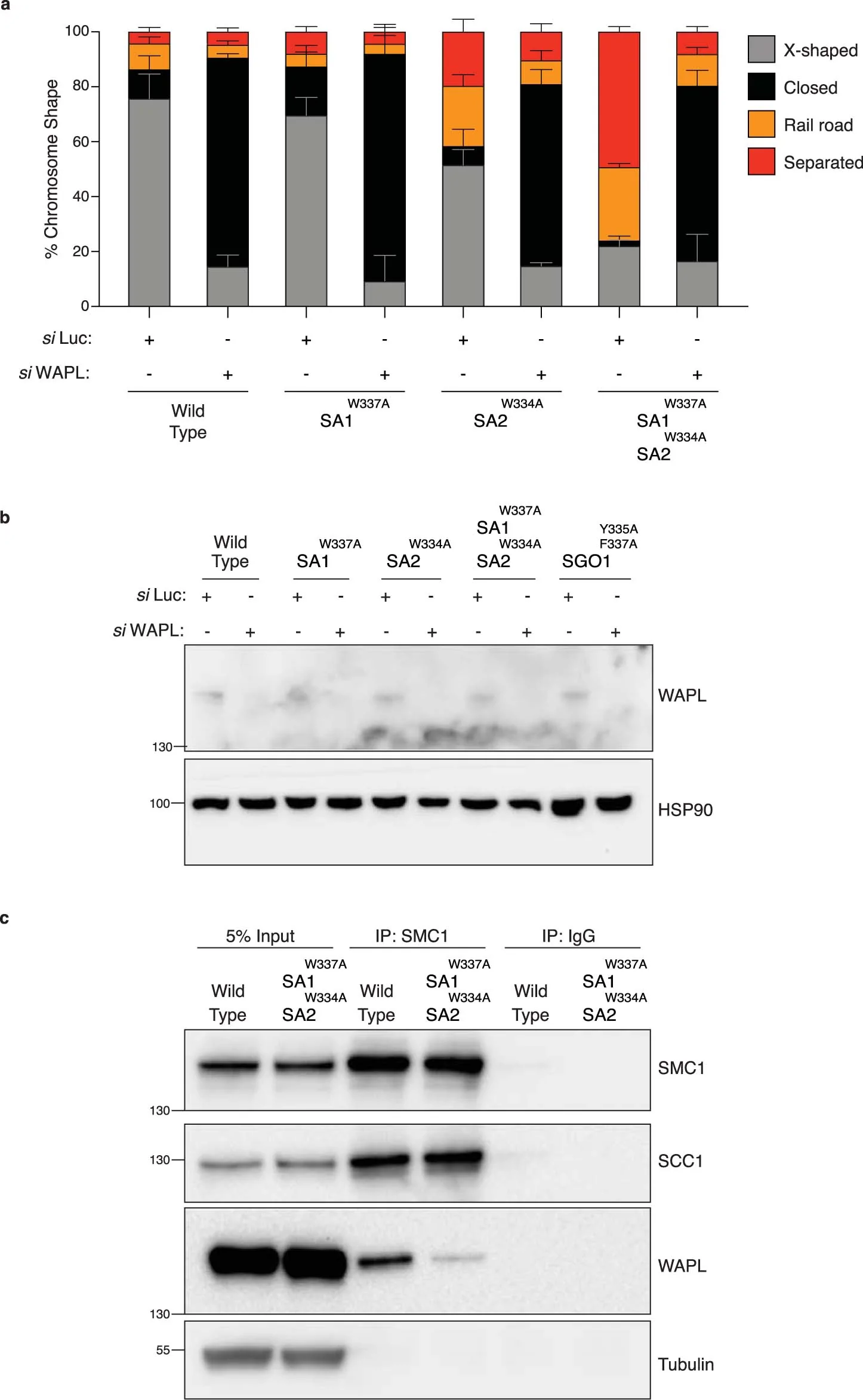
Cohesion defects of CES mutant cells are WAPL dependent. **a**,Quantification of cohesion phenotypes in prometaphase wild type, SA1 W337A, SA2 W334A, and SA1 W337A/SA2 W334A cells upon treatment with siLuciferase (siLuc) or siWAPL. Mean +/− SD of three independent experiments, with over 70 cells analysed per experiment. **b**, Representative western blot of wild type, SA1 W337A, SA2 W334A, SA1 W337A /SA2 W334A, and SGO1 Y335A/F337A cells treated with siLuciferase or siWAPL. HSP90 serves as a loading control. Experiment performed three independent times. **c**, Co-immunoprecipitation of WAPL to cohesin in wild type and SA1 W337A /SA2 W334A cells. Tubulin serves as loading control. Note that the SA mutants reduce, but to not fully abolish WAPL binding. This likely explains how WAPL can still release these mutant cohesin complexes from DNA. Experiment performed three independent times. Source data

Previous work has shown that SGO1 competes with WAPL for binding to the SA2 subunit of cohesin^18^. SGO1 could thereby protect against the WAPL-dependent prophase pathway of cohesin release. To investigate whether the SGO1–CES interaction in fact protects against this WAPL-mediated cohesin release, we tested whether WAPL depletion rescued the cohesion defects observed in cells with impaired SGO1–CES binding. WAPL depletion indeed rescued the cohesion defect observed in all cell lines that had impaired SGO1–CES binding, including SGO1^Y335A F337A^ cells, SA1^W337A^ and SA2^W334A^ cells, and SA1^W337A^ SA2^W334A^ double-mutant cells (Fig. 2f and Extended Data Fig. 7a,b). Coimmunoprecipitation experiments showed that WAPL binding to cohesin was only partially impaired in SA1^W337A^ SA2^W334A^ mutant cells (Extended Data Fig. 7c). Thus, competition with SGO1–CES interaction is a key but not the sole aspect of WAPL function, which presumably explains why SA1^W337A^ SA2^W334A^ mutant cells did not display an overcohesion phenotype. We conclude that the SGO1–CES interaction protects against a specific aspect of WAPL-mediated DNA release and thereby enables centromeric cohesion.

### The SGO1–CES interaction dictates SGO1 localization

**Fig. 3:**
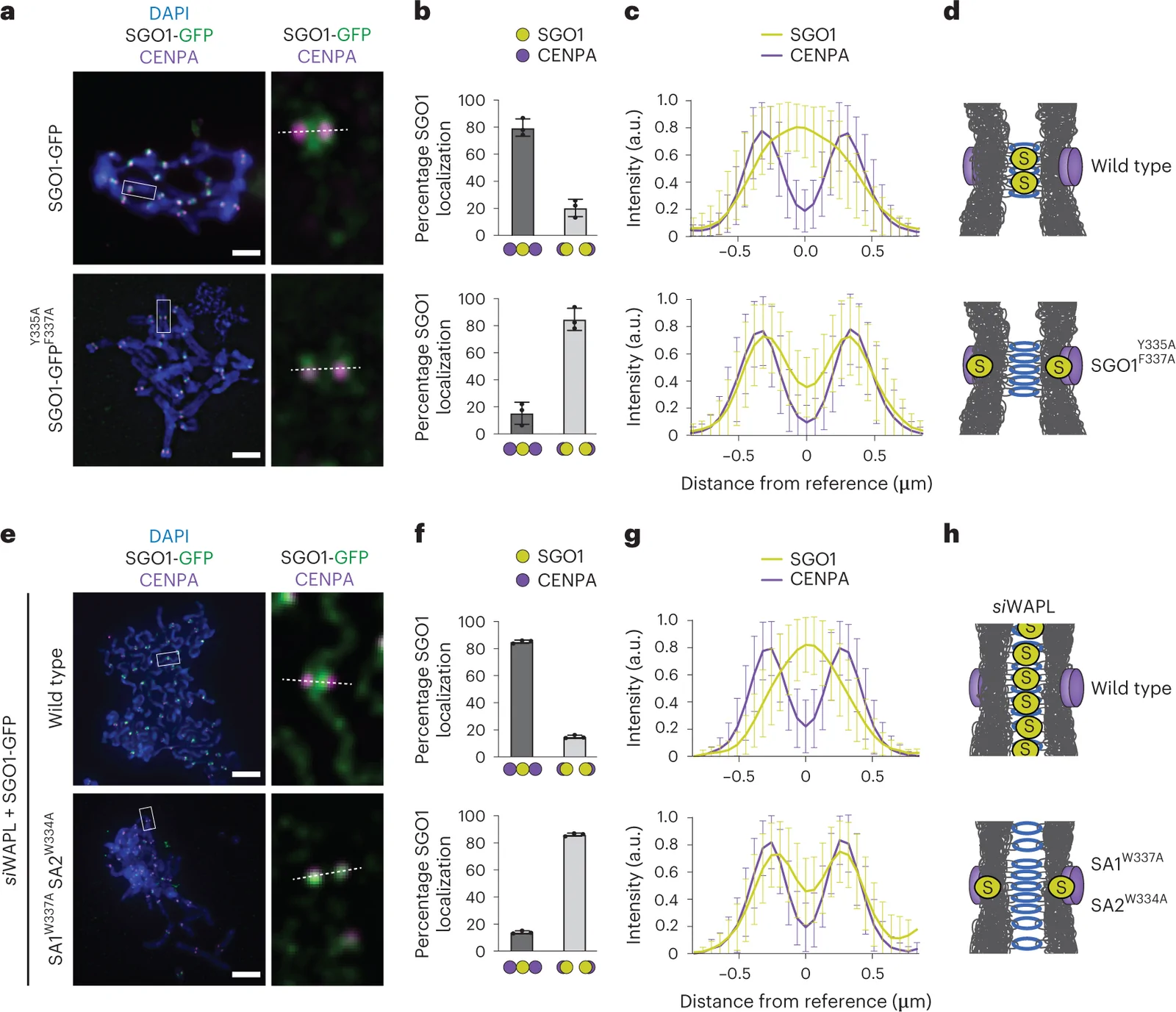
The SGO1–CES interaction dictates SGO1 localization. **a**, Representative immunofluorescence images of GFP-tagged wild-type SGO1 or SGO1^Y335A F337A^ (green) and CENPA (magenta) upon treatment with nocodazole. Scale bar, 5 μm. **b**, Quantification of centromeres with GFP signal enriched between CENPA signal of the two chromatids (dark gray column) or GFP signal enriched at the CENPA signal (light gray column) in cells transfected with GFP-tagged wild-type SGO1 or SGO1^Y335A F337A^. We analyzed four random centromeres over 30 cells. This experiment was performed three times; mean ± s.d. **c**, Quantification of the mean ± s.d. of the intensity of SGO1-GFP (yellow) and CENPA (magenta) along the centromeric region on cells treated with nocodazole. The intensity at each point was normalized to the highest intensity measured per chromosome. The point between two CENPA signals was established as the reference point for each measurement. We analyzed four random centromeres over 30 cells. This experiment was performed three times. **d**, Schematic representation of the predominant phenotype observed in (**a**–**c**). S, SGO1. **e**, Representative images of SGO1–GFP wild type (green) location with respect to CENPA (magenta) in WAPL-depleted HAP1 wild-type and SA1^W337A^ SA2^W334A^ cells upon nocodazole treatment. Scale bar, 5 μm. **f**, Quantification of the images depicted in **e**, using analysis methods as in **b**. We analyzed four random centromeres over 30 cells. This experiment was performed three times; mean ± s.d. **g**, Quantification of the images depicted in **e**, using analysis methods as in **c**. **h**, Schematic representation of the predominant phenotype observed in **e**–**g**. Source data

**Extended Data Fig. 8:**
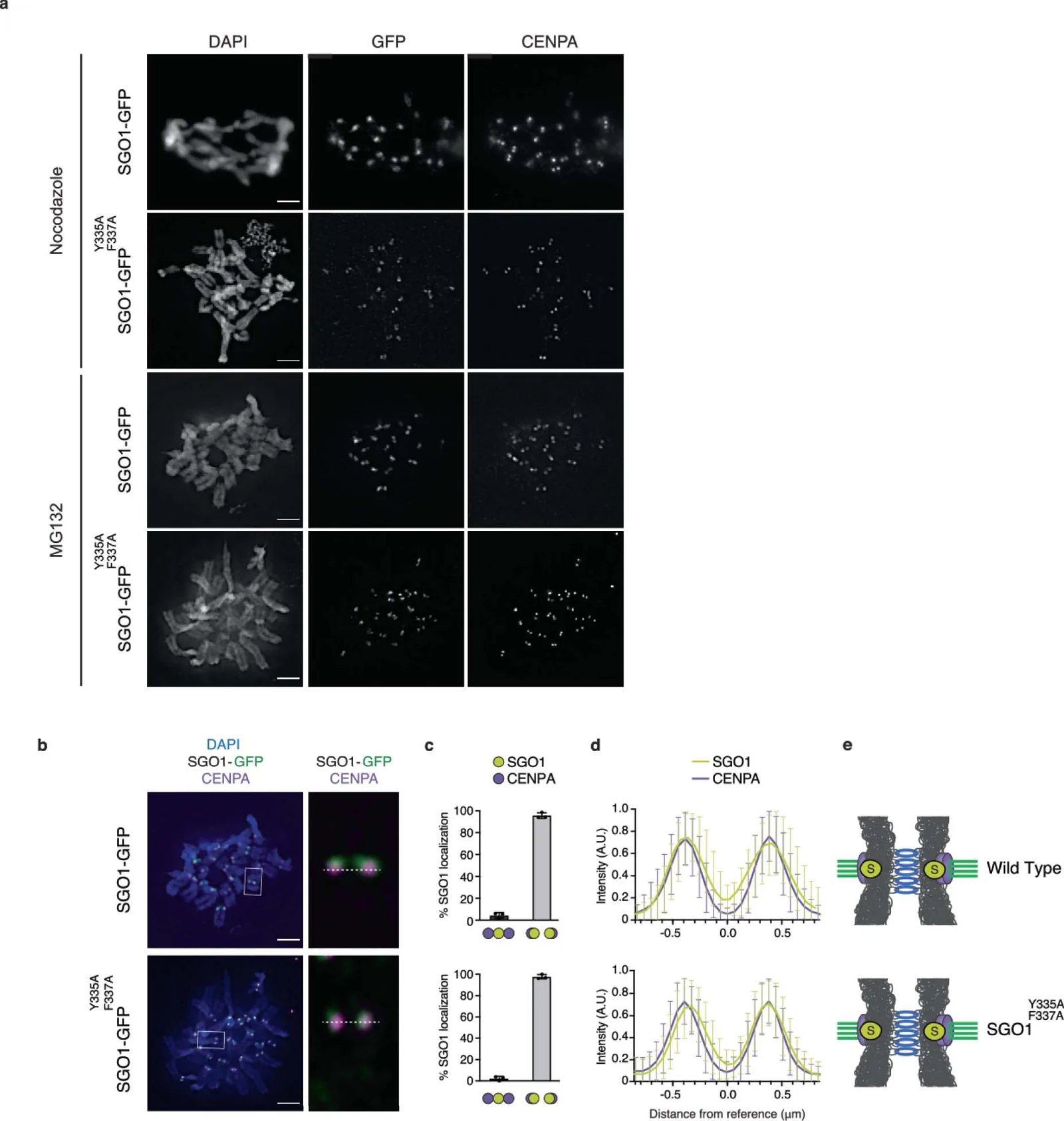
SGO1 localization in prometaphase and metaphase cells. **a**, Representative immunofluorescence images of SGO1-GFP or SGO1 Y335A/F337A-GFP (green), and CENPA (magenta) upon treatment with either nocodazole or MG132. Scale-bar: 5 μm. **b**, Merge of the representative images of GFP-tagged wild type SGO1 or SGO1 Y335A/F337A (green) and CENPA (magenta) upon treatment with MG132. Scale-bar: 5 μm. **c**, Quantification of centromeres with the GFP signal enriched in between the CENPA signal of the two chromatids (dark grey column) or with the GFP signal enriched at CENPA (light grey column), in MG132 treated cells transfected with GFP tagged wild type SGO1 or SGO1 Y335A/F337A. We analysed four random centromeres over 30 cells. This experiment was performed three times. Mean +/− SD. **d**, Quantification of the Mean +/− SD of the intensity of SGO1-GFP (yellow) and CENPA (magenta) along the centromeric region of cells treated with MG132. The intensity in each point was normalized to the highest intensity measured per chromosome. The point between two CENPA signals was established as the reference point for each measurement. We analysed four random centromeres over 30 cells. The experiment was performed three times. **e**, Schematic representation of the predominant phenotype observed in **(b**–**d)**. Source data

During prometaphase, SGO1 localizes to the inner centromere, where it protects cohesin. Upon proper microtubule–kinetochore attachment, SGO1 relocalizes towards the kinetochores^25^. To test whether the SGO1–CES interaction is involved in SGO1 localization, we transfected cells with a plasmid encoding a green fluorescent protein (GFP)-tagged SGO1 that was either wild type or harbored the SGO1^Y335A F337A^ mutation. We then scored for SGO1 localization by immunofluorescence chromosome spreads, comparing the absence versus the presence of microtubule–kinetochore attachments, using nocodazole- or MG132-treated cells, respectively. In nocodazole-treated cells, wild-type SGO1–GFP localized to the inner centromere as expected. The SGO1^Y335A F337A^ mutant, however, did not localize to the inner centromere and was primarily found at the kinetochores (Fig. 3a–d and Extended Data Fig. 8a). In MG132-treated cells, the localizations of wild-type SGO1 and the SGO1^Y335A F337A^ mutant were similar, in that both localized to kinetochores (Extended Data Fig. 8a–e).

**Extended Data Fig. 9:**
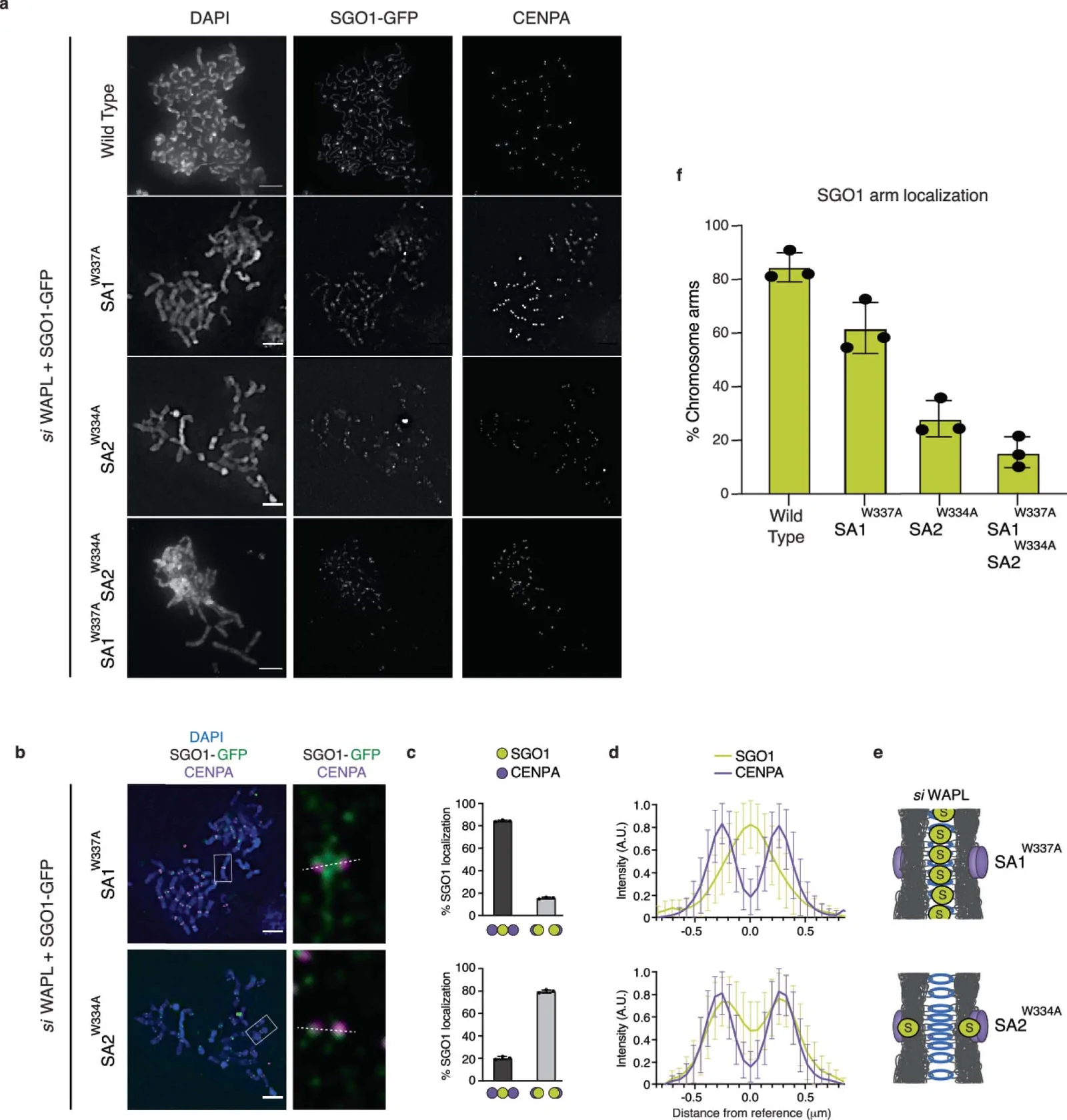
SGO1 localization in CES mutant cells. **a**, Representative images of SGO1-GFP (green) location with respect to CENPA (magenta) in WAPL-depleted wild type, SA1 W337A, SA2 W334A, and SA1 W337A/SA2 W334A cells upon treatment with nocodazole. Scale-bar: 5 μm. **b**, Merge of representative immunofluorescence images of GFP-tagged wild type SGO1 (green), and CENPA (magenta), in WAPL-depletion SA1 W337A and SA2 W334A mutant cells. Wild type and SA1/SA2 double mutant cells of this experiment are depicted in Fig. 3. Scale-bar: 5 μm. **c**, Quantification of centromeres with the GFP signal enriched in between the CENPA signal of both chromatids (dark grey column) or with the GFP signal enriched at CENPA (light grey column) in SA1 W337A, and SA2 W334A cells transfected with GFP tagged wild type SGO1. We analysed four random centromeres over 30 cells. This experiment was performed three times. Mean +/− SD **d**, Quantification of the Mean +/− SD of the intensity of SGO1-GFP (yellow) and CENPA (magenta) along the centromeric region on SA1 W337A, and SA2 W334A cells. The intensity in each point was normalized to the highest intensity measured per chromosome. The point between two CENPA signals was established as the reference point for each measurement. We analysed four random centromeres over 30 cells. The experiment was performed three times. **e**, Schematic representation of the predominant phenotype observed in **(b**–**d)**. **f**, Quantification of the different phenotypes of SGO1-GFP along chromosome arms of WAPL-depleted wild type, SA1 W337A, SA2 W334A, and SA1 W337A/SA2 W334A cells upon nocodazole treatment. Mean +/− SD of three independent experiments, with over 30 cells scored per condition per experiment. Source data

We then assessed the effects of the SA1^W337A^ and SA2^W334A^ mutations on SGO1 localization. To prevent secondary effects due to cohesion defects, we depleted WAPL using siRNAs. WAPL depletion indeed maintained cohesion in wild-type, SA1^W337A^, SA2^W334A^ and SA1^W337A^ SA2^W334A^ mutant cells (Extended Data Fig. 7a). Whereas SGO1 efficiently localized to the inner centromeres in wild-type and SA1^W337A^ cells following nocodazole treatment, this localization was lost in both SA2^W334A^ and SA1^W337A^ SA2^W334A^ mutant cells (Fig. 3e–h and Extended Data Fig. 9a–e). This result, together with the SGO1^Y335A F337A^ mutant data described above, shows that SGO1 localization to the inner centromere requires the SGO1–CES interaction, and that this predominantly involves the interaction with SA2.

At the start of mitosis, cohesin is localized along the entire length of chromosomes. The WAPL-dependent prophase pathway then removes cohesin along arms but not at centromeres. This change in cohesin localization corresponds with SGO1 localization^10,26^. To assess whether the SGO1–CES interaction plays a part in SGO1 localization to chromosome arms, we depleted WAPL to prevent prophase pathway cohesin release. In otherwise wild-type cells, this yielded a clear localization of SGO1 along the entire length of chromosomes. This phenotype was also present to a considerable degree in SA1^W337A^ mutant cells but less so in SA2^W334A^ and barely if at all in SA1^W337A^ SA2^W334A^ mutant cells (Fig. 3e and Extended Data Fig. 9b,f), again highlighting the key role of SA2 in SGO1 localization.

Together, these findings show that the SGO1–CES interaction has a vital role in SGO1 localization to chromosomes. Whereas SGO1 localization to kinetochores appears to be independently regulated, the SGO1–CES interaction, mainly through SA2, is a determinant of SGO1 localization to chromosome arms and inner centromeres. This latter interaction turns out to be key to centromeric cohesin protection.

## Discussion

**Fig. 4:**
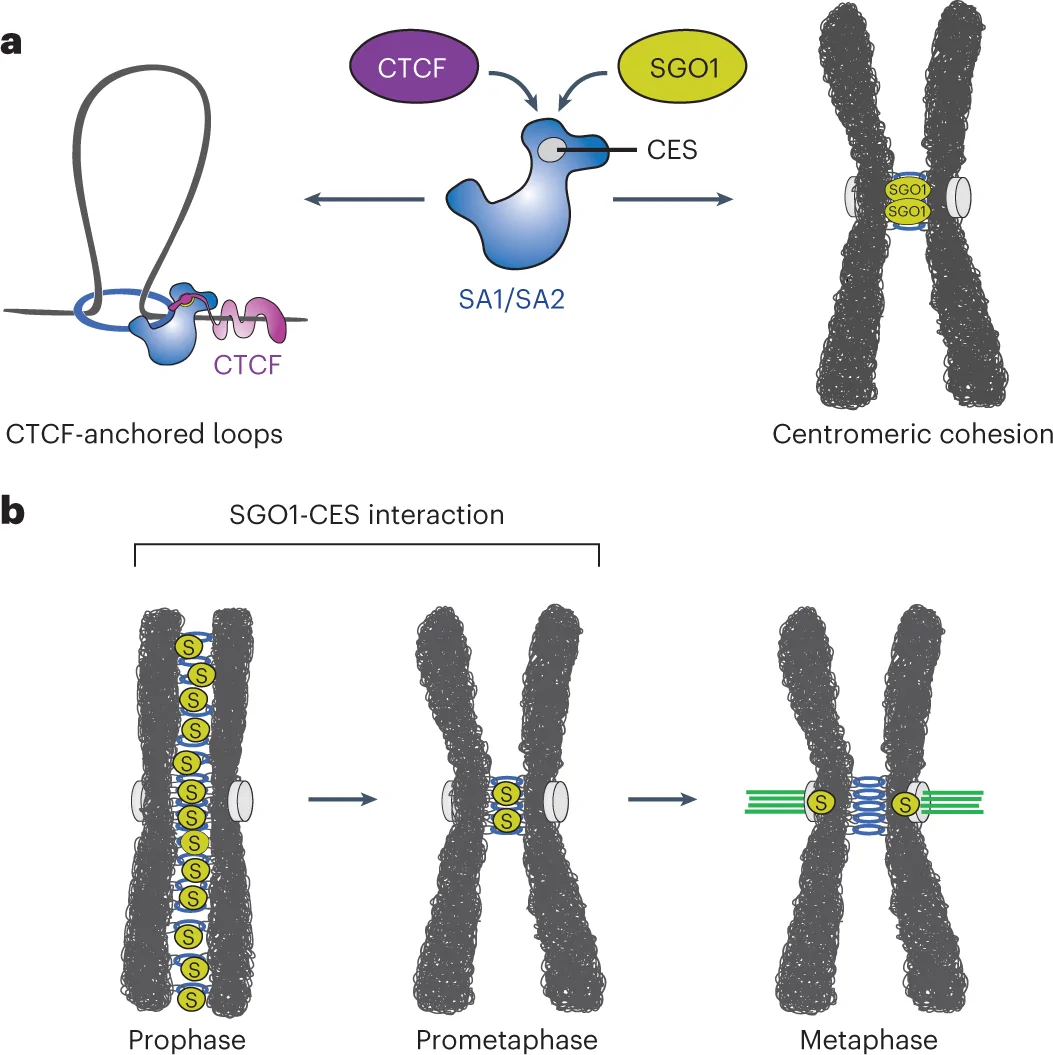
A universal mechanism to control cohesin complexes. **a**, Different chromosomal regulators control cohesin through a shared mechanism. CTCF through its YxF motif binds cohesin’s CES (gray circle) to stabilize CTCF-anchored loops^21^. SGO1 uses its YxF motif to bind cohesin’s CES to protect centromeric sister chromatid cohesion. Adapted with permission from ref. ^2^, Elsevier; ref. ^21^, Springer Nature Ltd. **b**, The SGO1–CES interaction dictates SGO1 localization along chromosomes until the establishment of microtubule–kinetochore attachments (S:SGO1).

In this study, we present the first structure of the interaction between SGO1 and cohesin. This interaction involves the binding of the YxF motif of SGO1 to the conserved CES interface of cohesin. This SGO1–CES interaction is very similar to the manner in which the architectural factor CTCF binds to cohesin. We build on previous work, which suggested that SGO1 interacts with the CES^18^, and we reveal that SGO1 does so by using its YxF motif. Both SGO1 and CTCF thus turn out to bind to the same CES interface in cohesin, and they do so by using their respective YxF motifs. Although SGO1 and CTCF appear to bind to cohesin in very similar manners, they control very different chromosomal processes. Disruption of the CTCF-CES interaction led to a dramatic change in the three-dimensional genome, through the loss of CTCF-anchored loops^21^. We now find that disruption of the SGO1–CES interaction, by contrast, leads to a dramatic cohesion defect. It thus appears that cohesin complexes are controlled through a universal mechanism, irrespective of whether these complexes build DNA loops or hold together the sister DNAs (Fig. 4a). Both DNA looping and cohesion are tightly regulated and are involved in processes ranging from DNA replication to transcription, repair and recombination. We should therefore consider the scenario where different chromosomal regulators involved in these processes may each employ CES binding to direct cohesin to control different chromosomal processes. The replicative helicase subunit MCM3 has for example been proposed to likewise bind cohesin^21^^,27^, which may control processes such as cohesion establishment. As such, SGO1 and CTCF may merely be the tip of the iceberg.

As we find that disruption of SGO1–CES interaction prevents localization of SGO1 to mitotic chromosomes at all sites except kinetochores, this suggests a model for SGO1 localization throughout mitosis. At the start of mitosis, SGO1 would then bind cohesin along chromosomes through interaction with the CES of cohesin. SGO1 is subsequently recruited to centromeres, presumably as a consequence of H2A phosphorylation by the centromeric kinase Bub1 (refs. ^28–30^). Here, SGO1 through CES binding protects centromeric cohesin from the WAPL-dependent prophase pathway. Upon establishment of bipolar microtubule attachment, SGO1 then relocates towards kinetochores^25^ (Fig. 4b). The SGO1–CES interaction thus plays a vital part in SGO1 localization through mitosis, is key to the protection of centromeric cohesion, ensures faithful chromosome segregation and thereby maintains a stable karyotype.

## Methods

### Genome editing and cell culture

HAP1 cells were cultured in Iscove’s modified Dulbecco’s medium (Invitrogen), containing 10% fetal bovine serum (Clontech), 1% UltraGlutamin (Lonza) and 1% penicillin–streptomycin (Invitrogen). Mutant cells were generated by CRISPR–Cas9 technology. Guide RNAs targeting exon 6 of SGO1 (primer, 5′-TGATGCTTACAATTTTAATT-3′), exon 10 of STAG1 (5′- TTGGCTGGACTCTTCATGAC-3′) and exon 11 of STAG2 (5′-GACAGTTATTTAAAATATGT-3′) were annealed into pX330. To mutate the locus of interest, we cotransfected a 100–120 base pair repair oligonucleotide with the desired mutation as well as a silent mutation: for SGO1Y^335A F337A^ (5′-CAAAAAAAAATGCACAAATCTGTCAGTTCCAATGATGCTGCCAATGCTAATTTGGAAGAAGGTGTTCATCTTACTCCTTTCCGACAAAAAGTGAGCAATG-3′), STAG1^W337A^ (5′-AGTACTGAGACAAACATAACTTCCATCAAAGCTTAGAACAG AGTAACTTACCCTGTCGTGAAGAGTAGCGCCAACATATTTTAGGTAACTGTCATTTAGGAAGGCATCACTATACATTTTCATC-3′) and STAG2^W334A^ (5′-CTTAATGACAGTTATTTAAAATATGTTGGTGCGACTATGCATGATAAGGTAAGATGTGCCCTTCAGACTGCTTCTTTCTATACATCGGCGTGGCTGTCTGCACCTCTCATTCATGAG-3′). We cotransfected pBabePuro at a ratio of 1:10 to the pX330 plasmid. Cells were treated with 2 μg μl^−1^ puromycin for 2 days for selection. Colonies were picked, genomic DNA of clones was isolated and mutations were validated by Sanger sequencing.

### siRNA transfection

All siRNAs were manufactured by Dharmacon (ON-TARGETplus). For SGO1 and luciferase we used SMARTpools, and for WAPL we used the following sequence: 5′-CAACAGUGAAUCGAGUAAUU-3′. Transfection was performed with 20 μM per siRNA final concentration, using Invitrogen RNAiMAX (Life Technologies), following the manufacturer’s instructions.

### Chromosome spreads

Cells were transfected with the corresponding siRNAs, and after 2 days the cells were treated with nocodazole as described previously^31^. Images were randomized by a homemade ImageJ macro and then visually assigned their corresponding phenotype. A parametric two-tailed *t* test was used to compare the scoring of cohesion phenotypes.

### Immunofluorescence

For immunofluorescence, cells were treated with nocodazole, fixed and stained as described previously^31^. For immunofluorescence spreads, cells were treated with the corresponding siRNA. After 24 h, cells were transfected using FuGENE transfection reagent (Promega) with 0.8 μg SGO1–GFP plasmid (kindly provided by S. Lens) or a SGO1^Y335A F337A^–GFP mutant plasmid. One day later, cells were treated with nocodazole for 1.5 h or with MG132 for 2 h, and mitotic cells were collected by shake-off. Cells were washed once in phosphate-buffered saline, followed by a quick spin onto microscope slides with a Shandon Cytospin centrifuge. Cells were extracted with PBS containing 0.3% Triton-X for 5 min and fixed in 4% paraformaldehyde for 15 min. The coverslips were washed three times with PBS containing 0.1% Triton-X before being incubated with antibodies at a 1:1000 dilution in PBS containing 3% BSA and 0.1% Triton-X overnight at 4 °C. Secondary antibody incubations were performed by incubation at room temperature for 1 h with DAPI in PBS containing 3% BSA and 0.1% Triton-X. Coverslips were mounted in Prolong Gold (Invitrogen).

Images were obtained using a DeltaVision deconvolution microscope (Applied Precision), and images were acquired using Softworx (Applied Precision) and ImageJ. To establish levels of SGO1 in prometaphase cells, we used an ImageJ macro that allowed us to calculate the level of SGO1 relative to CENPA. To identify the location of SGO1–GFP in mitotic cells, we first blinded the channel corresponding to GFP to prevent bias towards a phenotype. Next, we drew a straight line on four random chromosomes that showed two distinct centromeres and obtained the plot profile of both CENPA and GFP for each location.

### Live-cell imaging

Cells were grown on glass-bottomed dishes (LabTek). To visualize the DNA, 2 h before imaging, a SiR-DNA probe (1:2000, Spirochrome) was added. Images were taken using a DeltaVision deconvolution microscope (Applied Precision). Cells were imaged every 5 min using a ×40 air objective with 4 × 2.5 μm Z stacks. Images were acquired using Softworx (Applied Precision) and ImageJ.

### Fluorescence in situ hybridization

Prometaphase samples cells were obtained as described above. Fixed cells were dropped on cover slides and then dried. We added probes against the centromere of chromosome 8 (XCE 8 ORANGE, MetaSystems Probes) and shielded the cells with a coverslip and rubber cement. The slides were incubated for 2 min at 75 °C, followed by overnight incubation at 37 °C. The cells were washed with 0.4× SSC at 72 °C for 2 min, followed by washing at room temperature with 2× SSC, 0.05% Tween-20, for 30 s. The slides were washed with water and stained with DAPI, followed by mounting with Prolong Gold (Invitrogen).

G2 samples were collected by treating the cells for 18 h with RO-3306. We verified that the cells were synchronized in G2 by incubation in Nicoletti buffer followed by flow cytometry (BD LSRFortessa). Plots were generated with FlowJo (v.10). G2-synchronized cells were spun down and resuspended with fixative solution (methanol/acetic acid, 3:1), followed by the same protocol as described above.

Images were taken using a DeltaVision deconvolution microscope (Applied Precision), and images were acquired using Softworx (Applied Precision) and ImageJ. The fluorescence signal was categorized as singlet (distance between the two highest intensity signals ≤300 nm) or doublet (distance between the two highest intensity signals >300 nm), as described previously^32^.

### Immunoblotting and coimmunoprecipitation

Immunoblot and coimmunoprecipitation were performed as previously described^33^.

### Antibodies

The following antibodies were used as primary antibodies for immunofluorescence microscopy: SGO1 (SAB1405371, Sigma Aldrich), GFP (ab290, Abcam) and CENPA (07–574, Millipore; and ab13939, Abcam). For immunoblotting, the following primary antibodies were used: SA1 (ab4457, Abcam), SA2 (A300-158a, Bethyl Laboratories), SMC1 (A300-055A, Bethyl Laboratories), SCC1 (05-908, Millipore), WAPL (A-7, sc-365189, Santa Cruz), Sororin (ab192237, Abcam), HSP90 (sc-13119(F-8), Santa Cruz) and α-tubulin (T5168, Sigma Aldrich). All primary antibodies were used at a 1:1000 dilution with the exception of HSP90 and α-tubulin (1:10000). For coimmunoprecipitation, we used 4.5 μg of SMC1 (A300-055A, Bethyl Laboratories) or IgG (2729 S, Cell Signaling) per sample. Secondary antibodies were used at a 1:1000 dilution. For immunofluorescence microscopy we used: Alexa Fluor 488 goat anti-mouse (A-11001, Life Technology), Alexa Fluor 568 goat anti-mouse (A-11004, Life Technology), Alexa Fluor 488 goat anti-rabbit (A-11008, Life Technology) and Alexa Fluor 568 goat anti-rabbit (A-11011, Life Technology). For western blots, we used the following secondary antibodies: anti-goat-PO (P0449, DAKO), anti-rabbit-PO (P0448, DAKO) and anti-mouse-PO (P0447, DAKO).

### Constructs, protein expression and purification

SA2 amino acid residues 80–1060 were expressed as a GST fusion protein and SCC1 amino acid residues 281–420 as an N-terminally 6×His-tagged fragment as described previously^21^. Expression and purification were done as described previously^21^. SGO1 constructs were cloned into the *Bam*HI and *Not*I sites of pGEX-6P1. Mutagenesis was done using a Q5 Site-Directed Mutagenesis Kit (New England Biolabs). All proteins were expressed in *Escherichia coli* BL21 (DE3) by autoinduction, and purification was done as described previously^21^.

### Crystallization and structure determination

Crystallization of the SA2–SCC1 complex was done as described previously^18,21^. Crystals were soaked for 7 days with a 500 μM peptide solution including SGO1 amino acid residues 331–341 SNDAYNFNLEE. Crystals were cryoprotected as described previously^21^. Diffraction data were collected at 100 K at an X-ray wavelength of 0.9687 Å at beamline ID30A-1/MASSIF-1 (ref. ^34^) of the European Synchrotron Radiation Facility, with a Pilatus3 2M detector, using automatic protocols for the location and optimal centering of crystals^35^.

Data were processed with XDS^36^ and imported into CCP4 format using AIMLESS^37^. The structure was determined by molecular replacement using Phaser (Phenix 1.14-3260)^38^. A final model was produced by iterative rounds of manual model-building in Coot (COOT 0.8.0-3)^39^ and refinement using PHENIX (1.14-3260)^40^. The SA2–SCC1–SGO1 model was refined to a resolution of 3.2 Å with *R*_work_ and *R*_free_ values of 25% and 28%, respectively (Table 1). Structures were rendered with PyMOL (2.2.3). Analysis with MolProbity (4.3)^41^ showed that there were no residues in disallowed regions of the Ramachandran plot, and the all-atom clash score was 12.3 (63rd percentile). The computational model shown in Fig. 1c was calculated using AlphaFold v.2.1.1 with multimer model v1 weights^42^. The computational model shown in Fig. 2b was generated by superposition of an AlphaFold model for SA1 onto SA2 in the SA2–SCC1–SGO1 complex.

### GST pulldowns

GST pulldowns were done as described previously^21^ with small modifications. Briefly, 50 μM GST-tagged SGO1 constructs were mixed in 50 μl buffer 1 (20 mM Tris-HCl, pH 7.8, 500 mM NaCl, 0.5 mM TCEP, 0.1% Tween-20) containing 25 μl of a 50% slurry of GST Sepharose beads (Cytiva) per reaction. GST beads were incubated for 1 h at 4 °C, followed by four washes with 500 μl of buffer 1. Then, 2.5 μM of SA2–SCC1 was added, followed by overnight incubation at 4 °C. A 25-μl volume of the reaction was withdrawn as the reaction input, and the remainder was washed five times with 500 μl of buffer 1. Samples were boiled in 1× sodium dodecyl sulfate (SDS) sample loading buffer (New England Biolabs) for 5 min to obtain the bound fraction, followed by SDS polyacrylamide gel electrophoresis analysis. ITC was performed as described previously^21^. ITC data were analyzed with Origin 7.0.

### Statistics and reproducibility

No statistical method was used to predetermine the sample size. No data were excluded from the analyses. All experiments with phenotype calling were randomized, and the SGO1 signal was blinded in all experiments for SGO1 localization with respect to the centromere. Data were visualized with Prism 9. For all pairwise comparisons, we performed *t* test analyses, with a probability threshold of *P* = 0.05. GST pulldowns were repeated at least three times with consistency.

### Reporting summary

Further information on research design is available in the Nature Portfolio Reporting Summary linked to this article.

## Online content

Any methods, additional references, Nature Portfolio reporting summaries, source data, extended data, supplementary information, acknowledgements, peer review information; details of author contributions and competing interests; and statements of data and code availability are available at https://doi.org/10.1038/s41594-023-00968-y.

## Supplementary information

Supplementary Information Supplementary Fig. 1 FACS gating strategy.

Reporting Summary

## Source data

Source Data Fig. 3 Statistical source data.

Source Data Extended Data Figs. 2, 3, 5, 6, 8 and 9 Statistical source data.

Source Data Extended Data Fig. 4 Unprocessed western blots.

Source Data Extended Data Fig. 6 Unprocessed western blots.

Source Data Extended Data Fig. 7 Unprocessed western blots.

## Data Availability

All data and materials generated during this investigation are available upon request from the corresponding authors. Crystal structure coordinates are available from the Protein Data Bank under accession number 7ZJS. Source data are provided with this paper.
