# Supplementary material for: Structural basis of centromeric cohesion protection
Source: Nat Struct Mol Biol. 2023 Apr 20;30(6):853–9. doi: 10.1038/s41594-023-00968-y (PMC10279526; doi:10.1038/s41594-023-00968-y)
Supplement: Supplementary file 1 — Supplementary Fig. 1 FACS gating strategy. [file 41594_2023_968_MOESM1_ESM.pdf]

nature structural & molecular biology

Article

https://doi.org/10.1038/s41594-023-00968-y

Structural basis of centromeric cohesion protection

In the format provided by the authors and unedited

a

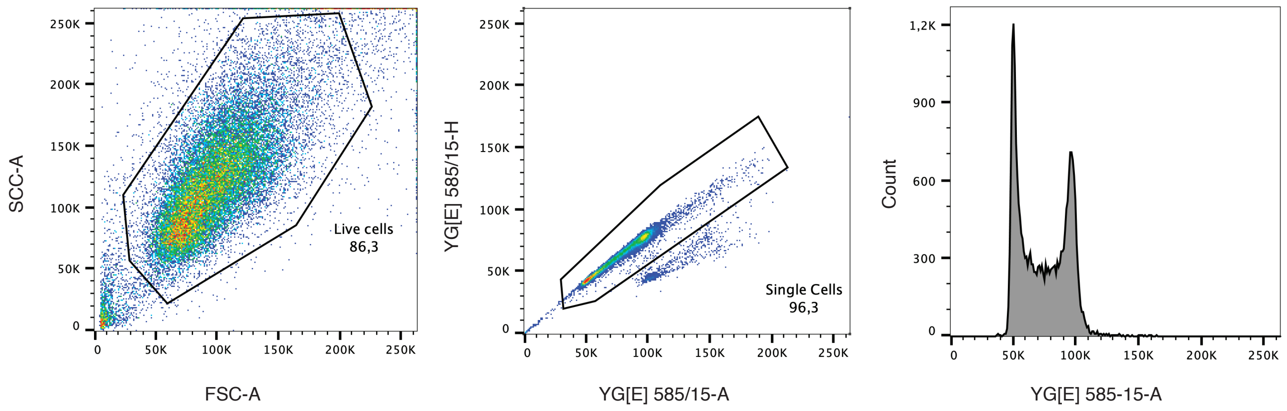

Supplementary Figure 1. FACS gating strategy.
a, FACS gating strategy for generation of DNA content histograms as used in in Extended Data Figure 6. Live cells were gated based on FCS/SCC (left), and single cells were gated based on 585/15-A and 585/15-H (middle). Histogram shows the DNA content per cell (585/15-A and count) (right).
