# Supplementary material for: Structural basis of centromeric cohesion protection
Source: Nat Struct Mol Biol. 2023 Apr 20;30(6):853–9. doi: 10.1038/s41594-023-00968-y (PMC10279526; doi:10.1038/s41594-023-00968-y)
Supplement: Supplementary file 2 — Reporting Summary [file 41594_2023_968_MOESM2_ESM.pdf]

nature portfolio

Corresponding author(s): Daniel Panne & Benjamin Rowland

Last updated by author(s): 14/02/2023

Reporting Summary

Nature Portfolio wishes to improve the reproducibility of the work that we publish. This form provides structure for consistency and transparency in reporting. For further information on Nature Portfolio policies, see our Editorial Policies and the Editorial Policy Checklist.

Statistics

For all statistical analyses, confirm that the following items are present in the figure legend, table legend, main text, or Methods section.

n/a	Confirmed	
☐	☒	The exact sample size (*n*) for each experimental group/condition, given as a discrete number and unit of measurement
☐	☒	A statement on whether measurements were taken from distinct samples or whether the same sample was measured repeatedly
☐	☒	The statistical test(s) used AND whether they are one- or two-sided<br>*Only common tests should be described solely by name; describe more complex techniques in the Methods section.*
☒	☐	A description of all covariates tested
☒	☐	A description of any assumptions or corrections, such as tests of normality and adjustment for multiple comparisons
☐	☒	A full description of the statistical parameters including central tendency (e.g. means) or other basic estimates (e.g. regression coefficient) AND variation (e.g. standard deviation) or associated estimates of uncertainty (e.g. confidence intervals)
☐	☒	For null hypothesis testing, the test statistic (e.g. F, t, r) with confidence intervals, effect sizes, degrees of freedom and P value noted<br>*Give P values as exact values whenever suitable.*
☒	☐	For Bayesian analysis, information on the choice of priors and Markov chain Monte Carlo settings
☒	☐	For hierarchical and complex designs, identification of the appropriate level for tests and full reporting of outcomes
☒	☐	Estimates of effect sizes (e.g. Cohen's d, Pearson's r), indicating how they were calculated

Our web collection on statistics for biologists contains articles on many of the points above.

Software and code

Policy information about availability of computer code

Data collection	Chromosome spreads were acquired using the Metafer system (Metasystems). Chromosome spreads images were blindly randomized by a in-house written ImageJ macro. Immunofluorescent and FISH images were taken using a Deltavison deconvolution microscope (Applied Precision) and image acquisition was done using Softworx (Applied Precision). Analysis of the mean SGO1 intensity in prometaphase cells was performed using an in-house written ImageJ macro.
Data analysis	Molecular replacement was done with Phaser (Phenix 1.14-3260).<br>Structure refinement was done with Phenix (1.14-3260). Computational models were calculated using AlphaFold v2.1.1 Structure building was done with COOT 0.8.0-3, Structure renderings were done with Pymol (2.2.3), Structure analysis was done with MolProbity (4.3), Gel band quantification was done with imageJ (1.8.0_112), ITC data were analyzed with Origin 7.0. Graphical representation and analysis was done using Prism 9. For data analysis in Fig. We used ImageJ 1.52p

For manuscripts utilizing custom algorithms or software that are central to the research but not yet described in published literature, software must be made available to editors and reviewers. We strongly encourage code deposition in a community repository (e.g. GitHub). See the Nature Portfolio guidelines for submitting code & software for further information.

Data

Policy information about availability of data

All manuscripts must include a data availability statement. This statement should provide the following information, where applicable:

- Accession codes, unique identifiers, or web links for publicly available datasets
- A description of any restrictions on data availability
- For clinical datasets or third party data, please ensure that the statement adheres to our policy

Coordinates for the SA2-SCC1-SGO1 complex are available from the PBD (PDB ID 7ZJS). Any other relevant data are available from the corresponding authors upon reasonable request.

Human research participants

Policy information about studies involving human research participants and Sex and Gender in Research.

Reporting on sex and gender	N/A
Population characteristics	N/A
Recruitment	N/A
Ethics oversight	N/A

Note that full information on the approval of the study protocol must also be provided in the manuscript.

Field-specific reporting

Please select the one below that is the best fit for your research. If you are not sure, read the appropriate sections before making your selection.

☒ Life sciences ☐ Behavioural & social sciences ☐ Ecological, evolutionary & environmental sciences

For a reference copy of the document with all sections, see nature.com/documents/nr-reporting-summary-flat.pdf

Life sciences study design

All studies must disclose on these points even when the disclosure is negative.

Sample size	For biochemical experiments, population sample size statistics do not apply. For Figures showing GST-pulldown analyses (Fig.1, Extended Data Fig. 1), appropriate controls are used to compare binding side-by-side. Wherever statistics have been derived, the number of repeat measurements and their consistency is mentioned in the figure legends.
Data exclusions	No data was excluded from the analysis.
Replication	We have indicated the number of repeat measurements made and consistency of the results obtained in the figure legends. All attempts the results were successful.
Randomization	For phenotype calling experiments were randomized.
Blinding	Investigators were blinded to allocation during experiments and outcome assessment.

Reporting for specific materials, systems and methods

We require information from authors about some types of materials, experimental systems and methods used in many studies. Here, indicate whether each material, system or method listed is relevant to your study. If you are not sure if a list item applies to your research, read the appropriate section before selecting a response.

Materials & experimental systems

n/a	Involved in the study
☐	☒ Antibodies
☐	☒ Eukaryotic cell lines
☒	☐ Palaeontology and archaeology
☒	☐ Animals and other organisms
☒	☐ Clinical data
☒	☐ Dual use research of concern

Methods

n/a	Involved in the study
☒	☐ ChIP-seq
☐	☒ Flow cytometry
☒	☐ MRI-based neuroimaging

Antibodies

Antibodies used

The following antibodies were used as primary antibodies for immunofluorescence microscopy: SGO1 (SAB1405371, Sigma Aldrich), GFP (ab290, Abcam), and CENP-A (07-574, Millipore, and ab13939, Abcam). For immunobloting the following primary antibodies were used: SA1 (ab4457, Abcam), SA2 (A300-158a, Bethyl laboratories), SMC1 (A300-055A, Bethyl Laboratories), SCC1 (05-908, Millipore), WAPL (A-7, sc-365189, Santa Cruz), Sororin (ab192237, Abcam), HSP90 (sc-13119(F-8), Santa Cruz), and tubulin (T5168, Sigma Aldrich). For coimmunoprecipitation we used SMC1(A300-055A, Bethyl Laboratories) or IgG (2729S, Cell Signaling). Secondary antibodies were used at a 1:1000 dilution. For immunofluorescence microscopy we used: Alexa FlourTM 488 goat anti-mouse, Alexa FlourTM 568 goat anti-mouse, Alexa FlourTM 488 goat anti-rabbit, and Alexa FlourTM 568 goat anti-rabbit (Life Technology). For western blots, we used the following secondary antibodies: anti-goat-PO and goat anti-mouse-PO (DAKO).

Validation

SGO1 : https://www.sigmaaldrich.com/NL/en/product/sigma/sab1405371
GFP: https://www.abcam.com/gfp-antibody-ab290.html
CENP-A: https://www.merckmillipore.com/NL/en/product/Anti-CENP-A-Antibody,MM_NF-07-574
CENP-A: https://www.abcam.com/cenpa-antibody-3-19-chip-grade-ab13939.html
SA1: https://www.abcam.com/sa1-antibody-ab4457.html
SA2: https://www.fortislife.com/products/primary-antibodies/goat-anti-sa2-antibody/BETHYL-A300-158
WAPL: https://www.scbt.com/p/wapl-antibody-a-7
HSP90: https://www.scbt.com/p/hsp-90alpha-beta-antibody-f-8
SMC1: https://www.thermofisher.com/antibody/product/SMC1-Antibody-Polyclonal/A300-055A
Sororin: https://www.abcam.com/cdca5-antibody-epr16331-c-terminal-ab192237.html
Tubulin: https://www.sigmaaldrich.com/NL/en/product/sigma/t5168

Eukaryotic cell lines

Policy information about cell lines and Sex and Gender in Research

Cell line source(s)

HAP1 wild type from Carette et al., Nature 2011 gift from the authors.
HAP1 SGO1Y335A/F337A, SA1 W337A and SA2 W334A generated in this study in HAP1 wild type background cells using CRISPR/Cas gene editing. SA1 W337A/SA2 W334A generated generated in this study in a HAP1 SA2 W334A background cells using CRISPR/Cas gene editing.

Authentication

Karyotyping. Point mutations were authenticated by Sanger sequencing

Mycoplasma contamination

All the cell lines were confirmed negative for mycoplasma contamination

Commonly misidentified lines (See ICLAC register)

No commonly misidentified line was used

Flow Cytometry

Plots

Confirm that:

☐ The axis labels state the marker and fluorochrome used (e.g. CD4-FITC).

☒ The axis scales are clearly visible. Include numbers along axes only for bottom left plot of group (a 'group' is an analysis of identical markers).

☐ All plots are contour plots with outliers or pseudocolor plots.

☒ A numerical value for number of cells or percentage (with statistics) is provided.

Methodology

Sample preparation

Cells were incubated in Nicoletti buffer

Instrument

Cell were analyzed by flow cytometry (BD LSRFortessaTM).

Software	Plots were generated with FlowJo (v.10)
Cell population abundance	We did not quantify the abundance of the G2 population
Gating strategy	Cells were gated into single cells and plotted in a histogram

☒ Tick this box to confirm that a figure exemplifying the gating strategy is provided in the Supplementary Information.
