# Supplementary figures and images for: Structural basis of centromeric cohesion protection
Source: Nat Struct Mol Biol. 2023 Apr 20;30(6):853–9. doi: 10.1038/s41594-023-00968-y (PMC10279526; doi:10.1038/s41594-023-00968-y)

Extended Data Fig. 4.

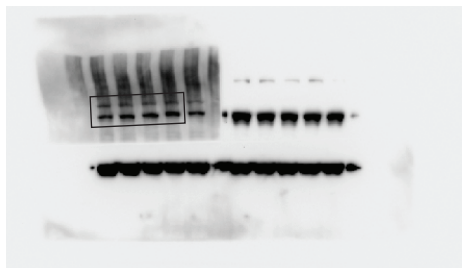

Goat-anti-SA1

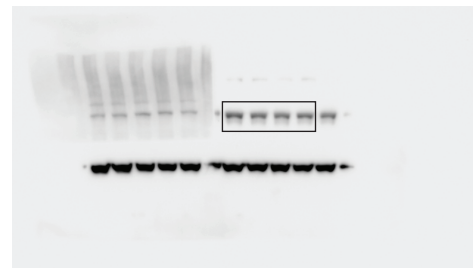

Goat-anti-SA2

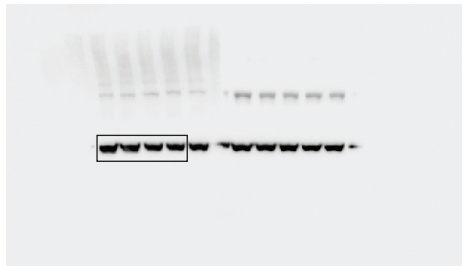

Mouse-anti-Hsp90

Supplement: Source Data Extended Data Fig. 4 — Unprocessed western blots. [file 41594_2023_968_MOESM5_ESM.pdf]

Extended Data Fig. 6.

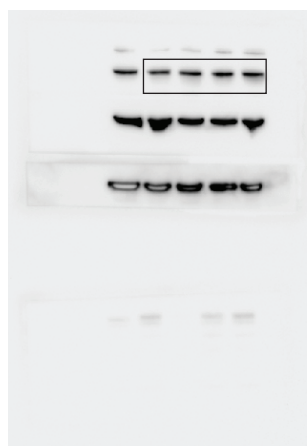

Rabbit-anti-SMC1

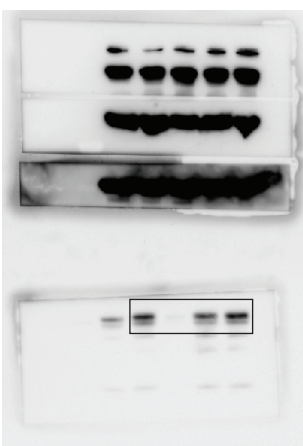

Rabbit-anti-Sororin

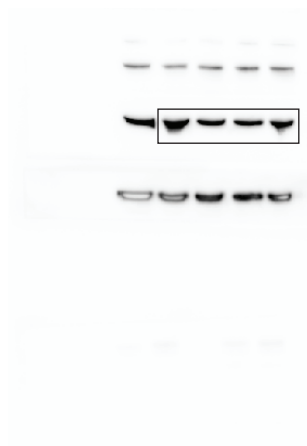

Mouse-anti-Hsp90

Supplement: Source Data Extended Data Fig. 6 — Unprocessed western blots. [file 41594_2023_968_MOESM6_ESM.pdf]

Extended Data Fig. 7.

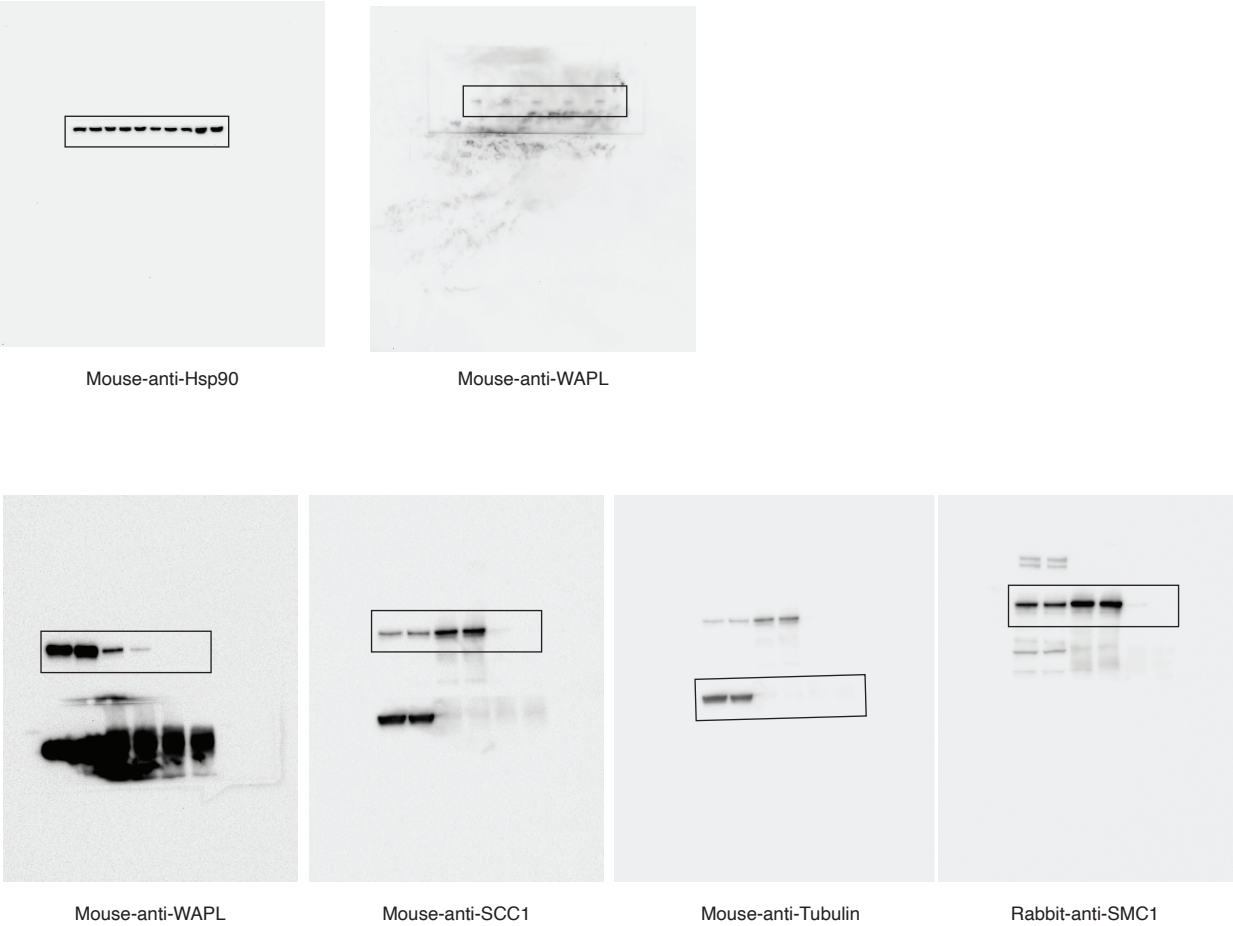

Supplement: Source Data Extended Data Fig. 7 — Unprocessed western blots. [file 41594_2023_968_MOESM7_ESM.pdf]
